# Association of Early Blood Pressure Levels and Outcomes in Ischemic Stroke Treated With Intravenous Thrombolysis: A Prospective Cohort Study

**DOI:** 10.1111/cns.70318

**Published:** 2025-03-12

**Authors:** Luyi Zhu, Jiali Xie, Qingjian Xie, Yiting Xu, Yinuo Chen, Yaojia Li, Junwei Zhang, Chunyang Pang, Lingfei Gao, Huan Yu, Binbin Deng

**Affiliations:** ^1^ Department of Neurology First Affiliated Hospital of Wenzhou Medical University Shanghai China; ^2^ Department of Neurology, Shanghai East Hospital Tongji University School of Medicine Shanghai China; ^3^ First Clinical College of Wenzhou Medical University Hangzhou China; ^4^ Department of Hepatobiliary and Pancreatic Surgery, the First Affiliated Hospital Zhejiang University School of Medicine Wenzhou China; ^5^ Department of Pediatrics Second Affiliated Hospital and Yuying Children's Hospital of Wenzhou Medical University Wenzhou China; ^6^ Department of Rehabilitation First Affiliated Hospital of Wenzhou Medical University Wenzhou China

**Keywords:** acute ischemic stroke, blood pressure, intracerebral hemorrhage, rt‐PA, thrombolysis

## Abstract

**Background and Purpose:**

Current guidelines for acute ischemic stroke (AIS) treatment recommend a lenient upper blood pressure (BP) threshold of 185/110 mmHg. However, stricter BP control has been reported to improve prognosis. This study aims to identify the optimal BP range following thrombolysis.

**Methods:**

This observational study included 340 AIS patients treated with rt‐PA thrombolysis at the First Affiliated Hospital of Wenzhou Medical University from December 2017 to December 2021. BP levels 24 h after thrombolysis were analyzed to determine their association with clinical outcomes. BP parameters included mean BP, variability (standard deviation (SD)), and decreased magnitudes. The primary outcome was the 90‐day modified Rankin Scale (mRS) scores.

**Results:**

Higher mean systolic BP (SBP) was associated with poorer outcomes, with adjusted odds ratios (aORs) of 1.25 (95% CI, 1.03–1.51), 1.23 (1.01–1.49), and 1.25 (1.02–1.52) per 10 mmHg increase within 0–2 h, 2–6 h, and 6–24 h post‐thrombolysis, respectively, but not for BP variability and decrease magnitudes. Significant improvements in outcomes were observed when the mean SBP was maintained within the range of 120–140 mmHg during both the 0–2 and 2–6 h periods, with aORs of 0.12 (95% CI, 0.02–0.75) and 0.19 (0.04–0.82), respectively. Larger decreases in SBP within 6 h post‐thrombolysis were associated with a lower risk of intracerebral hemorrhage. These findings were consistent across subgroups and sensitivity analyses.

**Conclusions:**

Achieving sustained low SBP levels (120–140 mmHg within the first 6 h) over 24 h is linked to better outcomes in thrombolyzed AIS patients.

## Graphical abstract

A cohort of 340 AIS patients treated with rt‐PA thrombolysis was analyzed. BP at 39 time points showed significant disparities in 24‐h post‐thrombolysis systolic BP between mRS 0–2 and mRS 3–6 groups, with lower systolic BP indicating better 90‐day functional outcomes.

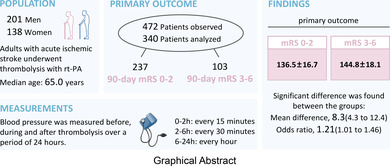

## 1 Introduction

Intravenous recombinant tissue plasminogen activator (rt‐PA) thrombolysis is an effective treatment for acute ischemic stroke (AIS) [1, 2]. Early hemodynamic management after thrombolysis is critical for prognosis [3]. Although elevated blood pressure (BP) is a common phenomenon after AIS [4], it is often associated with poor outcomes [5]. Current guidelines recommend maintaining BP below 185/110 mmHg before, during, and after thrombolysis [6]. Nevertheless, there is no consensus on the optimal BP targets for improving functional prognosis during the peri‐thrombolysis period. Some studies suggest that intensive BP lowering may enhance outcomes [7, 8]. However, the Enhanced Control of Hypertension and Thrombolysis Stroke Study, a randomized controlled trial (RCT) in AIS thrombolysis patients, did not show significant improvements in outcomes with intensive BP lowering (targeting systolic BP (SBP) 130–140 mmHg within 1 h) compared to standard lowering (150–180 mmHg), despite a significant reduction in hemorrhagic events [9]. In addition to mean BP [7, 8, 10, 11], BP variability (BPV) and reduction amplitude have garnered increasing attention, with higher variability [12, 13, 14, 15] and smaller reductions [12, 14] associated with increased hemorrhage [10, 12, 13, 14, 15] and worse outcomes [12, 13, 14]. However, studies on optimal BP ranges and the timing of BP intervention are lacking, and it is likely that the impact of BP on outcomes varies over time [16, 17]. Thus, our study divided the peri‐thrombolysis period into distinct stages, defined by key time points. BP parameters, including mean BP, variability (standard deviation (SD)), and both absolute and relative decrease magnitudes (ΔBP, R BP), were analyzed. Our primary objective was to assess the relationships between these BP parameters and the occurrence of intracranial hemorrhage (ICH) within the first 24 h, as well as functional outcomes at 90 days.

## 2 Methods

### 2.1 Study Design

A prospective longitudinal cohort study was conducted at the First Affiliated Hospital of Wenzhou Medical University from December 2017 to December 2021, focusing on patients with AIS treated with thrombolysis, from admission to post‐discharge follow‐up. During this period, all suspected AIS patients underwent a computed tomography (CT) scan upon arrival at the emergency department to rule out intracerebral hemorrhage. Following the evaluation of guideline‐recommended indications and contraindications, rt‐PA thrombolysis was administered to eligible patients. Patients who underwent additional thrombectomy were excluded from the study. Continuous ECG monitoring was provided for thrombolysis patients, and their BP was measured and recorded before, during, and after thrombolysis. After the completion of thrombolysis, patients were transferred from the emergency department to the ward for further treatment. Clinical trajectories during hospitalization were meticulously documented. Post‐discharge outcomes were assessed and recorded through telephone follow‐ups conducted by experienced clinicians.

### 2.2 Patients

Our cohort study analyzed data from 472 consecutive AIS patients who received rt‐PA thrombolysis treatment at the First Affiliated Hospital of Wenzhou Medical University between December 2017 and December 2021 (Figure S1). Patient follow‐up continued for 3 months to assess modified Rankin Scale (mRS) scores. After excluding patients with missing mRS scores at the 90‐day follow‐up (*n* = 98), including one patient who passed away from lung cancer (*n* = 1), as well as those who underwent endovascular thrombectomy (*n* = 23) and had insufficient BP measurements (*n* = 10), a total of 340 patients were included in the final analysis. The study protocol was approved by the Ethics Committee at the First Affiliated Hospital of Wenzhou Medical University and was conducted in accordance with the Helsinki Declaration.

### 2.3 Clinical Data and BP Parameters

The following baseline data were collected: (1) demographic characteristics (age and sex); (2) clinical characteristics (BP, National Institutes of Health Stroke Scale (NIHSS) score, antihypertensive treatment, onset‐to‐treatment time, rt‐PA dosage, stroke subtype); (3) medical history (history of diabetes, hypertension, hypercholesterolemia, prior stroke, atrial fibrillation, smoking and alcohol use); (4) comorbidities (pulmonary infection, urinary tract infection, deep vein thrombosis in the lower extremities, heart failure, liver dysfunction, renal dysfunction, extracranial hemorrhage, myocardial infarction, symptomatic epilepsy, and post‐stroke emotional disturbance).

BP data, including SBP and diastolic BP (DBP), were recorded during thrombolysis. The first BP measurement taken at the start of thrombolysis was labeled as “BP before IVT”. During the 60‐min rt‐PA infusion, BP was measured every 15 min, resulting in three pairs of BP readings defined as “BP during IVT”. Upon completion of thrombolysis, BP was recorded as “BP after IVT”. Subsequent measurements were taken every 15 min for the first 2 h, every 30 min from 2 to 6 h, and every hour from 6 to 24 h using a standard manual cuff sphygmomanometer.

BP parameters are defined as follows: mean BP represents the average of all BP measurements within a specific time period, while SD represents the SD. ΔBP denotes the absolute decrease in BP, calculated by subtracting the baseline BP from the mean BP of the corresponding period. The baseline BP for calculating the decrease during thrombolysis is the “BP before IVT,” while for post‐thrombolysis, it is the “BP after IVT.” R BP (relative decrease of BP) is calculated by dividing ΔBP by the corresponding baseline BP.

### 2.4 Outcomes

The primary outcome of our study was the 90‐day mRS score after thrombolysis, with a favorable outcome defined as an mRS score of 0–2. Secondary outcomes included the dichotomized 90‐day mRS score of 0–1 versus 2–6, as well as the occurrence of intracerebral hemorrhage (ICH) within 24 h following IVT. All mRS scores were assessed by qualified physicians who were blinded to the clinical data, using various methods such as telephone, email, questionnaires, and outpatient follow‐ups. The presence of ICH was determined via CT or magnetic resonance imaging (MRI), with interpretation performed by specialized radiologists.

### 2.5 Statistical Analysis

Data normality was assessed using the Shapiro–Wilk test, with a *p*‐value > 0.05 indicating a normal distribution. Normally distributed data were presented as mean ± SD and compared using *t*‐tests. The homogeneity of variance was evaluated using Levene's test. Skewed data were presented as the median and interquartile range (median, IQR) and analyzed using Mann–Whitney *U* tests. Categorical data were reported as frequency and percentage (*n*, %) and analyzed using chi‐squared tests.

The association between SBP parameters and the primary outcome was initially explored using locally estimated scatterplot smoothing (LOESS) to assess potential nonlinear trends. When indicated, regression models included quadratic or cubic terms. Linear relationships were examined using binary logistic regression, adjusting for confounders such as age, sex, admission NIHSS, history of atrial fibrillation, diabetes mellitus, stroke history, and smoking status. Odds ratios (ORs) and 95% confidence intervals (CIs) were reported per 10 mmHg increase for BP before IVT, BP after IVT, and mean BP, and per 10% increase for R BP. Median values of categorized SBP parameters were used as continuous variables to test for linear trends via binary logistic regression. Restricted cubic splines were employed to explore the relationships between ΔSBP, R SBP, and the occurrence of any ICH within 24 h after IVT.

Stratified analyses were conducted to investigate potential interactions by incorporating multiplicative interaction terms into the models, with likelihood ratio tests used for significance. Sensitivity analysis was performed by first excluding patients with fewer than 35 BP measurement time points. Further analysis included comorbidities in the model. Statistical significance was set at a two‐tailed *p*‐value < 0.05. All analyses and graphical representations were performed using SPSS version 27.0 (SPSS Inc., Chicago, IL, USA) and R version 4.2.2 (R Foundation for Statistical Computing, Vienna, Austria). The study adhered to the Strengthening the Reporting of Observational Studies in Epidemiology (STROBE) guidelines.

## 3 Results

### 3.1 Baseline Characteristics and 90‐Day Functional Outcome

The baseline characteristics of the included 340 patients treated with rt‐PA thrombolysis are summarized in Table 1. The mean age of the cohort was 65.0 years, with 40.7% being female. At the 90‐day follow‐up, 237 patients had an mRS score of 0–2, while 103 patients scored 3–6. Patients with poorer outcomes (mRS 3–6) had a significantly higher mean age of 69.5 years compared to those with favorable outcomes (mRS 0–2) (*p* < 0.001). No significant gender differences were noted between the two groups (*p* = 0.797). The group with poorer outcomes had a higher prevalence of hypertension, prior stroke, and atrial fibrillation history. Significant differences in baseline SBP and TOAST classification were also observed, with the poorer outcome group receiving antihypertensive treatment more frequently (all *p* < 0.05). Among comorbidities, pulmonary infection, deep vein thrombosis, and liver dysfunction were significantly associated with worse 3‐month outcomes.

**TABLE 1:** Baseline characteristics of IVT‐treated patients with 90‐day mRS 0–2 and mRS 3–6.

	Overall	mRS 0–2	mRS 3–6	*p*
	(*n* = 340)	(*n* = 237)	(*n* = 103)	
Demographic characteristics				
Age (median, IQR)	66, 57–74	64, 55–72	70, 61–79	< 0.001
Female sex, *n*, %	138, 40.7	95, 40.3	43, 41.7	0.797
Medical history				
Diabetes mellitus, *n*, %	79, 26.5	49, 23.8	30, 32.6	0.111
Hypertension, *n*, %	268, 84.8	177, 81.2	91, 92.9	0.008
Hypercholesterolemia, *n*, %	112, 47.9	74, 46.0	38, 52.1	0.387
Prior stroke, *n*, %	32, 9.5	15, 6.4	17, 16.7	0.003
Atrial fibrillation, *n*, %	68, 23.5	37, 18.6	31, 34.4	0.003
Tobacco use, *n*, %	134, 45	100, 47.8	34, 38.2	0.126
Alcohol use, *n*, %	94, 31.8	69, 33.3	25, 28.1	0.374
Current stroke event				
SBP before IVT, mean ± SD	151.6 ± 19.4	150.1 ± 18.7	155.1 ± 20.8	0.041
SBP after IVT, mean ± SD	148.2 ± 19.6	146.2 ± 19.3	153.0 ± 19.7	0.005
Baseline NIHSS (median, IQR)	5, 3–9	4, 2–6	10, 7–14	< 0.001
Antihypertensive treatment, *n*, %	118, 34.7	73, 30.8	45, 43.7	0.022
Minutes from stroke onset to IVT (median, IQR)	202, 158–240	205, 154–241	200, 162–240	0.722
rt‐PA dosage (median, IQR)	57, 50–63	57, 50–63	58, 50–63	0.600
Frequency of total BP measurements (median, IQR)	36, 34–38	36, 35–38	36, 34–38	0.272
TOAST subtypes, *n*, %				
Large artery atherosclerosis	149, 54.4	91, 49.2	58, 65.2	0.027
Cardioembolism	40, 14.6	26, 14.1	14, 15.7	
Small artery occlusion	44, 16.1	43, 23.2	1, 1.1	
Undetermined	41, 15.0	25, 13.5	16, 18.0	
Comorbidities				
Pulmonary infection, *n*, %	43, 12.6	16, 6.8	27, 26.2	< 0.001
Urinary tract infection, *n*, %	7, 2.1	4, 1.7	3, 2.9	0.753
Deep vein thrombosis, *n*, %	24, 7.1	12, 5.1	12, 11.7	0.029
Myocardial infarction, *n*, %	2, 0.6	1, 0.4	1, 1.0	0.515
Heart failure, *n*, %	6, 1.8	3, 1.3	3, 2.9	0.541
Liver dysfunction, *n*, %	13, 3.8	8, 3.4	5, 4.9	0.730
Renal dysfunction, *n*, %	43, 12.6	16, 6.8	27, 26.2	< 0.001
Extracranial hemorrhage, *n*, %	5, 1.5	3, 1.3	2, 2.0	1.000
Symptomatic epilepsy, *n*, %	4, 1.2	2, 0.8	2, 1.9	0.752
Post‐stroke emotional disturbance, *n*, %	4, 1.2	4, 1.7	0, 0	0.436

Abbreviations: DBP, diastolic blood pressure; IQR, Interquartile Range; IVT, intravenous thrombolysis; mRS, modified Rankin Scale; NIHSS, National Institutes of Health Stroke Scale; rt‐PA, recombinant tissue plasminogen activator; SBP, systolic blood pressure; SD, standard deviation.

**FIGURE 1:**
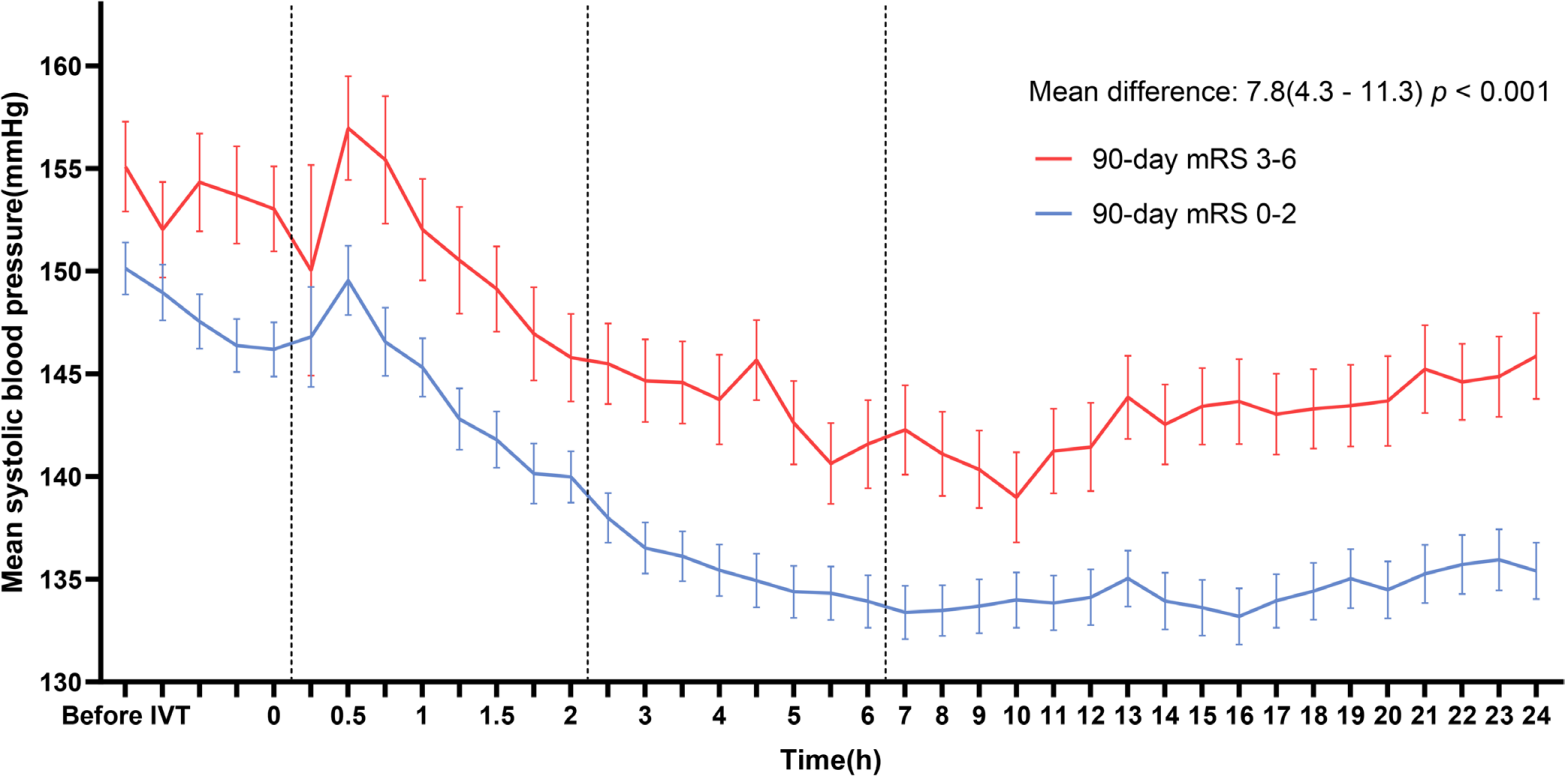
Mean SBP from pre‐thrombolysis to 24 h post‐thrombolysis. The average SBP data, accompanied by their corresponding standard errors, were recorded for both the 90‐day mRS 0–2 and 3–6 groups at each time point. SBP measurements were taken prior to thrombolysis, three times during thrombolysis, and at the end of thrombolysis. Subsequently, SBP was measured every 15 min for the first 2 h, every 0.5 h from 2 to 6 h, and hourly from 6 to 24 h. The mean difference in SBP between the two groups was 7.8 mmHg (95% CI 4.3–11.3). Abbreviations: IVT, intravenous thrombolysis; SBP, systolic blood pressure; mRS, modified Rankin Scale.

Figure 1 illustrates the SBP data at each measurement point, shown as mean and standard error (SE). A significant difference was observed between the two groups (*p* < 0.001), with a mean difference of 7.8 mmHg (95% CI: 4.3–11.3). Both groups experienced a notable increase in SBP during the first half‐hour post‐thrombolysis, likely due to the transfer from the emergency department to the neurology ward. Following this, SBP gradually declined and stabilized. The group with an mRS score of 3–6 maintained higher SBP levels throughout the 24‐h post‐thrombolysis period. Figure S2 further demonstrates significant hourly differences in SBP between the two groups. Additionally, no significant difference in the magnitude of BP decrease was found between patients who received antihypertensive medications and those who did not (Figure S3 and Table S1).

### 3.2 Mean BP and 90‐Day Outcomes

Table 2 demonstrates the linear relationships between SBP parameters and outcomes. Baseline SBP values, including both SBP before IVT and SBP after IVT, as well as the mean and SD of SBP during and after thrombolysis at various time intervals (0–2 h, 2–6 h, 6–24 h), exhibited significant linear correlations with the primary outcome (Table 2). Similar trends were observed for SBP parameters in relation to the 90‐day mRS scores of 2–6 (Table S2). After adjusting for potential confounders, mean SBP remained significantly associated with outcomes, with aORs per 10 mmHg increase of 1.25 (95% CI, 1.03–1.51; *p* = 0.023), 1.23 (95% CI, 1.01–1.49; *p* = 0.042), and 1.25 (95% CI, 1.02–1.52; *p* = 0.029) for the 0–2 h, 2–6 h, and 6–24 h post‐thrombolysis periods, respectively. Similar patterns of association were found for DBP and mean arterial pressure (MAP) (Tables S3 and S4). Predicted probabilities from the binary logistic regression models for SBP parameters and the primary outcome are illustrated in Figure S5.

**TABLE 2:** Association between outcomes and SBP parameters during IVT and different time periods after IVT.

Parameters	90‐day mRS 3–6				Any ICH within 24 h			
	Unadjusted		Adjusted^a^		Unadjusted		Adjusted^a^	
	OR	*p*	OR	*p*	OR	*p*	OR	*p*
SBP before IVT	1.15 (1.00–1.31)	0.043	1.19 (1.00–1.43)	0.053	0.84 (0.68–1.05)	0.117	0.81 (0.64–1.03)	0.089
SBP during IVT								
Mean^b^	1.18 (1.04‐1.35)	0.013	1.17 (0.98–1.40)	0.086	0.77 (0.61–0.97)	0.023	0.69 (0.53–0.90)	0.006
SD	1.04 (1.00–1.08)	0.080	1.05 (0.99–1.11)	0.091	1.00 (0.93–1.08)	0.912	0.98 (0.89–1.07)	0.646
△SBP	1.00 (0.98–1.01)	0.734	1.01 (0.98–1.03)	0.568	1.01 (0.98–1.04)	0.362	1.03 (0.99–1.06)	0.125
R SBP	0.93 (0.73–1.19)	0.585	1.08 (0.75–1.57)	0.685	1.31 (0.81–2.11)	0.266	1.54 (0.90–2.62)	0.114
SBP after IVT^b^	1.20 (1.05–1.37)	0.006	1.20 (1.00–1.44)	0.047	0.82 (0.66–1.02)	0.078	0.73 (0.57–0.94)	0.014
SBP t0‐2h								
Mean^b^	1.25 (1.08‐1.43)	0.002	1.25 (1.03–1.51)	0.023	0.97 (0.77–1.21)	0.758	0.89 (0.69–1.15)	0.379
SD	1.05 (1.01–1.09)	0.025	1.04 (0.99–1.09)	0.122	0.95 (0.87–1.03)	0.185	0.90 (0.83–0.99)	0.021
△SBP	1.00 (0.98–1.02)	0.960	1.00 (0.97–1.02)	0.710	0.97 (0.94–1.00)	0.047	0.95 (0.92–0.99)	0.013
R SBP^c^	0.99 (0.78–1.26)	0.951	0.96 (0.66–1.40)	0.823	0.65 (0.43–0.97)	0.033	0.51 (0.30–0.86)	0.011
SBP t2‐6h								
Mean^b^	1.35 (1.17‐1.56)	< 0.001	1.23 (1.01–1.49)	0.042	1.11 (0.88–1.38)	0.379	0.97 (0.74–1.26)	0.804
SD	1.09 (1.03–1.15)	0.003	1.04 (0.97–1.12)	0.301	1.00 (0.92–1.10)	0.946	0.94 (0.84–1.04)	0.234
△SBP	0.99 (0.98–1.01)	0.440	1.00 (0.98–1.02)	0.791	0.97 (0.94–0.99)	0.012	0.96 (0.93–0.99)	0.014
R SBP^c^	0.90 (0.73–1.11)	0.332	1.04 (0.76–1.43)	0.806	0.65 (0.47–0.90)	0.010	0.60 (0.39–0.92)	0.020
SBP t6‐24h								
Mean^b^	1.34 (1.17‐1.55)	< 0.001	1.25 (1.02–1.52)	0.029	1.00 (0.81–1.25)	0.992	0.88 (0.67–1.15)	0.339
SD	1.11 (1.04–1.19)	0.003	1.05 (0.96–1.16)	0.265	0.94 (0.84–1.06)	0.340	0.88 (0.75–1.02)	0.093
△SBP	1.00 (0.98–1.01)	0.562	1.00 (0.98–1.02)	0.756	0.98 (0.96–1.01)	0.121	0.98 (0.95–1.00)	0.087
R SBP^c^	0.91 (0.74–1.12)	0.370	1.03 (0.76–1.41)	0.833	0.74 (0.54–1.02)	0.066	0.70 (0.48–1.03)	0.069

Abbreviations: ICH, Intracerebral Hemorrhage; IVT, intravenous thrombolysis; mRS, modified Rankin Scale; OR, odds ratio; SBP, systolic blood pressure; SD, Standard Deviation.

^a^ Adjusted for age, sex, admission NIHSS, history of atrial fibrillation, history of diabetes mellitus, history of stroke, smoking status.

^b^ Odds ratio (OR) and 95% CI per 10 mmHg increase.

^c^ Odds ratio (OR) and 95% CI per 10% increase.

**FIGURE 2:**
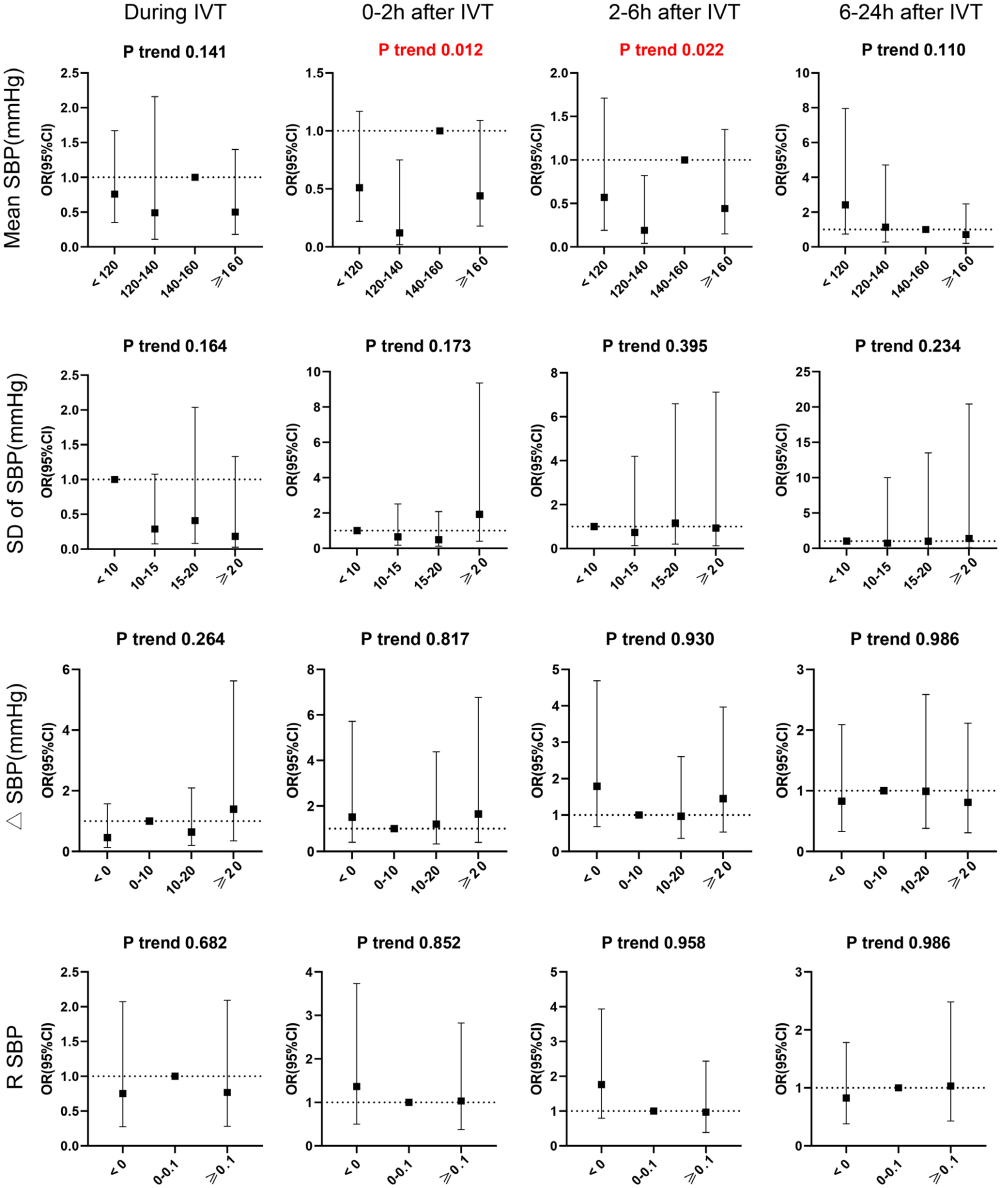
Association between categorized SBP parameters during IVT and different time periods after IVT and 90‐day mRS 3–6. The odds ratio (OR) and 95% confidence interval (CI) were calculated for each 10 mmHg increase in mean SBP and each 10% increase in R SBP. The analysis was adjusted for age, sex, admission NIHSS, history of atrial fibrillation, history of diabetes mellitus, history of stroke, and smoking status. △SBP, absolute decrease of systolic blood pressure; IVT, intravenous thrombolysis; R SBP, relative decrease of systolic blood pressureSBP, systolic blood pressure; SD, Standard Deviation.

**FIGURE 3:**
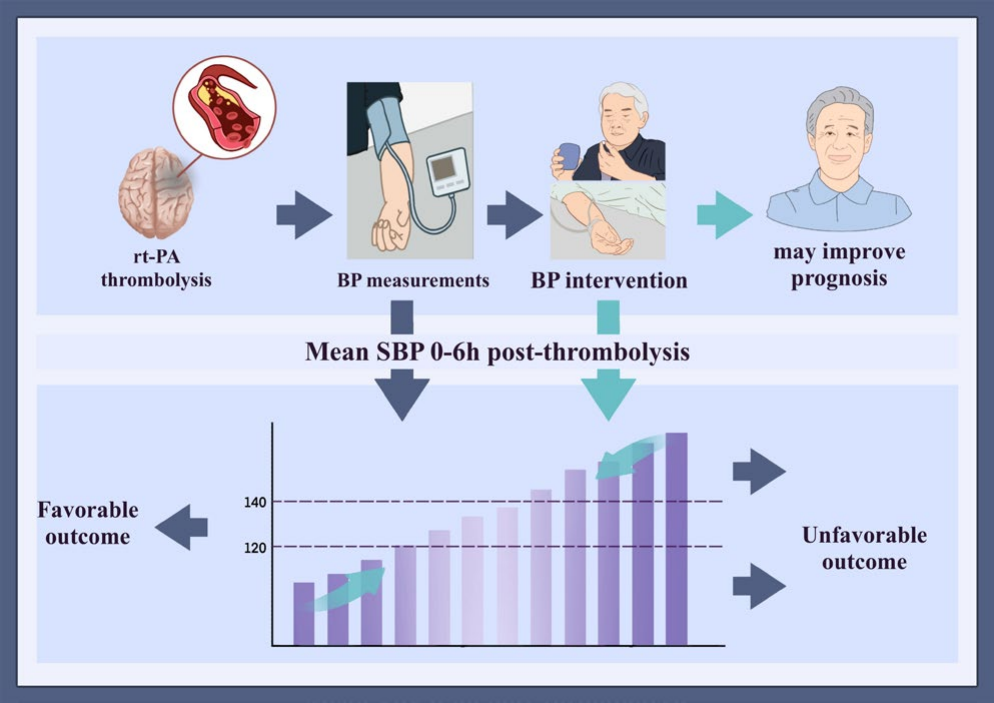
The schematic diagram of SBP during the first 6 h post‐thrombolysis as significant prognostic predictors and potential target for intervention. Rt‐PA, recombinant tissue plasminogen activator; SBP, systolic blood pressure.

Further analysis of categorized mean SBP and the primary outcome, using 140–160 mmHg as the reference, showed that lower SBP levels of 120–140 mmHg were associated with a reduced incidence of poor outcomes (Figure 2). The aORs per 10 mmHg increase in the 120–140 mmHg range were 0.12 (95% CI, 0.02–0.75; *p* trend, 0.012) at 0–2 h post‐thrombolysis and 0.19 (95% CI, 0.04–0.82; *p* trend, 0.022) at 2–6 h post‐thrombolysis (Table S5) No significant differences were observed for SBP levels below 120 mmHg or above 160 mmHg compared to the reference. Additionally, no significant associations were found for categorized SD, ΔSBP, or R SBP (all *p* trends > 0.05). Similar patterns were observed for the 90‐day mRS scores of 2–6 (Figure S6), indicating that maintaining an average SBP of 120–140 mmHg within the first 6 h post‐thrombolysis is associated with the most favorable 90‐day outcomes. This suggests that targeting an SBP range of 120–140 mmHg during this period may improve prognosis (Figure 3).

### 3.3 BP Decrease and ICH Within 24 h After IVT

Significant linear correlations were found between SBP decrease magnitudes and the occurrence of any ICH within 24 h after IVT (Table 2). Greater decreases in SBP were associated with a lower incidence of ICH within this timeframe. The aORs for ΔSBP during the 0–2 h and 2–6 h intervals were 0.95 (95% CI, 0.92–0.99, *p* = 0.013) and 0.96 (95% CI, 0.93–0.99, *p* = 0.014), respectively. Similarly, the aORs per 10% increase in R SBP during these intervals were 0.51 (95% CI, 0.30–0.86, *p* = 0.011) and 0.60 (95% CI, 0.39–0.92, *p* = 0.020), respectively. However, no significant associations were found between SBP decrease magnitudes and the primary outcome (all *p* > 0.05).

Further exploration of the relationship between SBP decrease and any ICH within 24 h post‐thrombolysis using restricted cubic splines revealed notable inflection points at a decrease magnitude of 0 across all time intervals (0–2 h, 2–6 h, 6–24 h) (Figure S7). The curves demonstrated steeper slopes after this inflection point, suggesting that the decrease in ICH risk associated with SBP lowering post‐thrombolysis is more pronounced compared to the increased risk observed with elevated SBP.

### 3.4 Stratified Analyses and Sensitivity Analyses

In subgroup analyses stratified by age (≥ 65 or < 65), SBP before IVT (< 140, 140–160, ≥ 160), history of atrial fibrillation (yes or no), and TOAST classification (large artery atherosclerosis or non‐large artery atherosclerosis), no significant interactions were observed between mean SBP and the primary outcome or mRS 2–6 (Figure S8 and Tables S6 and S7). Additionally, no interactions were found between ΔSBP and R SBP for the occurrence of any ICH within 24 h post‐IVT (Table S8).

In sensitivity analyses excluding patients with fewer than 35 BP measurement time points (Table S9), the results remained consistent with those presented in Tables 2 and S2, confirming the robustness of our findings. Furthermore, after additional adjustments for comorbidities, the results continued to show statistical significance (Table S10).

## 4 Discussion

Based on the analysis, the following conclusions can be drawn: (1) Higher mean BP, including SBP and MAP, within the first 24 h after thrombolysis was linked to worse 90‐day outcomes. Specifically, each 10 mmHg increase in mean SBP corresponded to an approximately 20% higher risk of achieving a mRS of 3–6 at 90 days. (2) For categorized mean SBP post‐thrombolysis, maintaining SBP between 120 and 140 mmHg during the first 6 h appeared to be the optimal range. SBP values below 120 mmHg or above 140 mmHg did not show significant differences in outcomes. (3) Although the group with poorer outcomes exhibited greater BP variability, this difference did not reach statistical significance. (4) Larger SBP decreases within the 0–2 and 2–6 h intervals post‐thrombolysis were associated with a lower risk of ICH within 24 h, but these decreases were not significantly linked to improved 90‐day functional outcomes.

Elevated BP is commonly observed after a stroke [4] and is considered a physiological response to the ischemic event [18]. In most cases, this elevation resolves naturally within a few days due to compensatory mechanisms and the improvement of ischemic injury [18, 19]. Additionally, thrombolysis‐induced reperfusion may accelerate the decrease in BP [20]. In our study, we observed a decreasing trend in BP within 24 h after thrombolysis, likely attributable to the revascularization effect of rt‐PA treatment and physiological compensation. Under normal conditions, cerebral autoregulation maintains stable cerebral blood flow (CBF) during systemic BP fluctuations. However, in AIS, this regulatory capacity is often impaired [21], potentially resulting in a linear relationship between CBF and cerebral perfusion pressure, with CBF depending on MAP. Maintaining higher BP in stroke patients can improve perfusion to the ischemic penumbra, potentially salvaging threatened tissue and limiting infarct expansion [22, 23]. Conversely, excessively high BP can lead to progressive constriction of small arteries, eventually causing passive vasodilation, increased CBF, disruption of the blood–brain barrier, and cerebral edema [24].

In our categorized SBP analysis, maintaining a mean SBP of 140–160 mmHg during the first 6 h appeared optimal for prognosis. Evidence suggests that aggressive SBP lowering does not significantly reduce perfusion in patients with impaired autoregulation [25, 26]. Previous studies have reported a U‐shaped or J‐shaped relationship between BP after rt‐PA thrombolysis and outcomes [7, 8], but were limited by fewer BP measurements and inconsistent conclusions. Our study addressed these limitations, offering novel insights into the complex relationship between BP and outcomes in the context of rt‐PA thrombolysis.

Worse outcomes were observed to be associated with higher BP in the 24 h following thrombolysis in AIS patients, according to our results. The European Cooperative Acute Stroke Study (ECASS) [27], ECASS 2 [28], and Safe Implementation of Thrombolysis in Stroke‐International Stroke Thrombolysis Register (SITS‐ISTR) [8] trials reported links between high BP post‐thrombolysis and unfavorable outcomes, aligning with our findings. Interestingly, while earlier research noted associations between high BP and poor outcomes, as well as increased ICH risk [29], we did not find a significant relationship between higher BP and ICH risk. This suggests that the adverse effects of BP increases on prognosis extend beyond ICH risk alone, highlighting the complexity of underlying mechanisms. Although not statistically significant, a trend toward higher mean BP before and during thrombolysis was observed in the poor outcome group. This indicates that BP after thrombolysis may better reflect arterial recanalization and collateral circulation status, potentially offering greater prognostic value than BP before and during thrombolysis.

Greater BPV was observed in the poor outcome group, though the difference was not statistically significant. A previous study noted that BPV was associated with worse outcomes in patients with failed recanalization, but no significant relationship was found in those with successful recanalization [30]. The absence of data on patients' recanalization status in our study, as well as in other studies lacking similar data [13, 28, 31], may explain the inconsistent findings regarding BPV and outcomes.

We found a significant association between larger SBP decreases within the 0–2 and 2–6 h periods post‐thrombolysis and lower ICH rates within 24 h. This association remained significant even after adjusting for post‐thrombolysis SBP. However, no association was found between SBP decreases and 90‐day outcomes. While ICH is a serious post‐thrombolysis complication and often a major contributor to poor outcomes [32], abrupt BP lowering over a short period can decrease CBF, potentially offsetting the benefits of reduced ICH. Further research is needed to better delineate this balance and develop optimal BP management strategies.

This study analyzed BP by considering three key aspects: mean BP, BPV, and decreased magnitudes. Despite extensive research on post‐thrombolysis BP management [3], the relationship between specific time intervals within the first 24 h post‐thrombolysis and outcomes remains poorly understood. Our study aimed to fill this gap by dividing the 24‐h period into three segments: 0–2 h, 2–6 h, and 6–24 h. Incorporating data from 39 time points allowed for a more comprehensive understanding of BP dynamics and outcomes following thrombolysis in AIS.

## 5 Limitations

Several limitations should be acknowledged. First, the data were derived from a single center in China, which may limit the generalizability of the findings to other populations. Second, although significant associations were observed between BP parameters and outcomes, this study does not demonstrate a direct improvement in outcomes through active BP control. Third, mechanistic investigations into factors contributing to poor 90‐day outcomes were lacking, as data on reperfusion, collateral circulation status, and imaging assessments of the ischemic penumbra and cerebral edema were unavailable.

## 6 Conclusion

Our study provides novel insights into the early monitoring of BP in patients undergoing rt‐PA thrombolysis, emphasizing the importance of closely monitoring BP within the first 24 h, particularly during the initial 6 h. Maintaining SBP between 120 and 140 mmHg during this period may improve 90‐day outcomes. Further confirmation of these findings through RCTs is warranted.

## Author Contributions

Luyi Zhu and Jiali Xie were responsible for data statistics and writing the paper. Yiting Xu, Yinuo Chen, Yaojia Li, Junwei Zhang, Chunyang Pang, Lingfei Gao, and Huan Yu collected data. Binbin Deng provided resources and designed the study.

## Ethics Statement

The Ethical Decision Committee of the Research Administration at First Affiliated Hospital of Wenzhou Medical University approved the study (KY2023‐R213). All patients agreed to participate in the study and use their clinic data and information for research purposes.

## Consent

All participants agreed to publications related to this study.

## Conflicts of Interest

The authors declare no conflicts of interest.

## Supporting information


Appendix S1.

## Data Availability

Data and material can be shared with the consent of the corresponding author.
