# Supplementary material for: Association of Early Blood Pressure Levels and Outcomes in Ischemic Stroke Treated With Intravenous Thrombolysis: A Prospective Cohort Study
Source: CNS Neurosci Ther. 2025 Mar 12;31(3):e70318. doi: 10.1111/cns.70318 (PMC11903326; doi:10.1111/cns.70318)
Supplement: Supplementary file 1 — Appendix S1. [file CNS-31-e70318-s001.docx]

**Supplementary Information**

Catlogue

Graphical abstract 3

Figure S1: Study recruitment flow chart. 4

Figure S2: Distribution of SBP at 24 time points after thrombolysis in the 90-day mRS 0-2 and 3-6 groups. 5

Figure S3 Box plots of Delta SBP during IVT and different time periods after IVT, stratified by the classes of antihypertensives 6

Figure S4: LOESS plot for association between SBP parameters and ordinal 90-day mRS. 7

Figure S5: Predicted probability of SBP parameters during IVT and different time periods after IVT on 90-day mRS 3-6. 8

Figure S6: Association between categorized SBP parameters during IVT and different time periods after IVT and 90-day mRS 2-6. 9

Figure S7: Restricted cubic spline models of reduction of SBP during IVT and different time periods after IVT and any ICH within 24h after IVT. 10

Figure S8: Subgroup analyses for the risk of 90-day mRS 3-6 by mean SBP during IVT and different time periods after IVT. 11

Table S1. Decreases in SBP in IVT-treated patients with and without blood pressure intervention 12

Table S2. Association between 90-day mRS 2-6 and SBP parameters during IVT and different time periods after IVT 13

Table S3. Association between outcomes and DBP parameters during IVT and different time periods after IVT 14

Table S4. Association between outcomes and MAP parameters during IVT and different time periods after IVT. 15

Table S5. Association between categorical mean systolic blood pressure with 90-day mRS 3-6 16

Table S6. Subgroup analyses for the risk of mRS 3-6 at 90 days by mean SBP during IVT and different time periods after IVT 17

Table S7. Subgroup analyses for the risk of mRS 2-6 at 90 days by mean SBP during IVT and different time periods after IVT 19

Table S8. Subgroup analyses for the risk of any ICH within 24h by △SBP and R SBP during different time periods after IVT 21

Table S9. Association between outcomes and SBP parameters during IVT and different time periods after IVT (timepoints of blood pressure measurements >= 35) 23

Table S10. Association between outcomes and SBP parameters during IVT and different time periods after IVT (additionally adjusted for comorbidity) 24


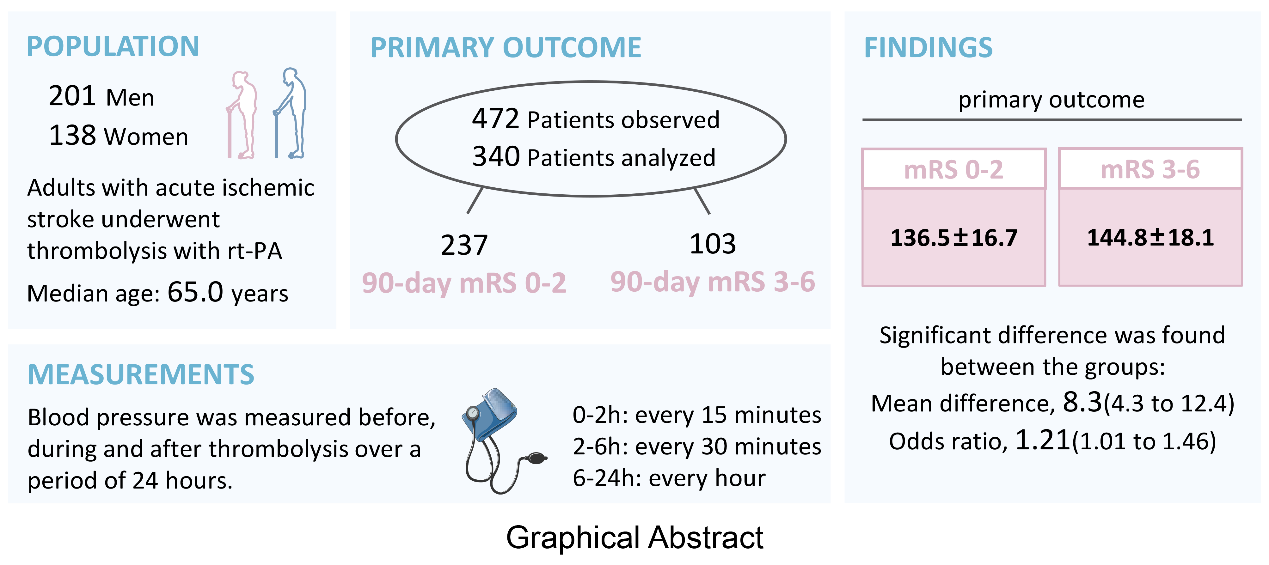


# Graphical abstract

**
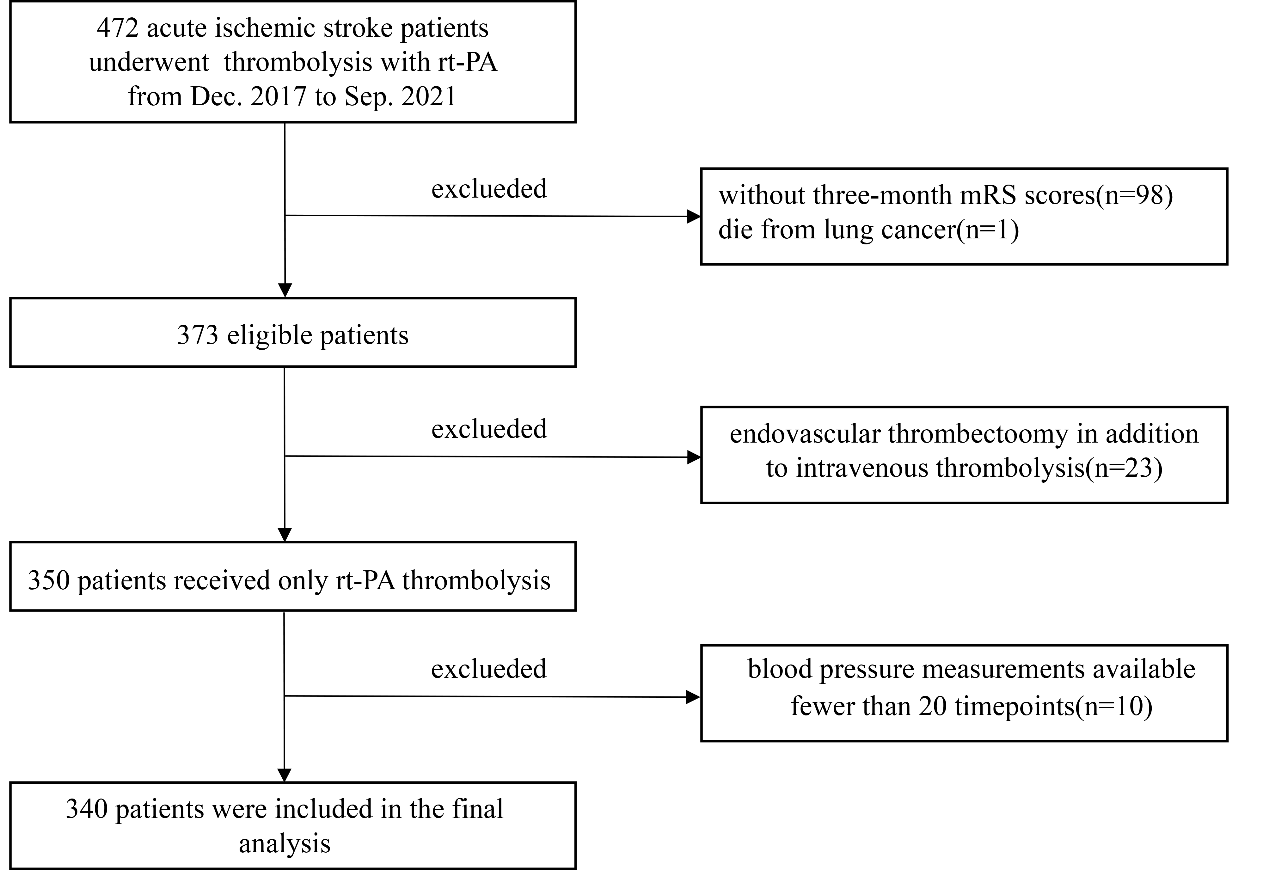
**

# Figure S1: Study recruitment flow chart.

Abbreviations: mRS, modified Rankin Scale; rt-PA, recombinant tissue plasminogen activator


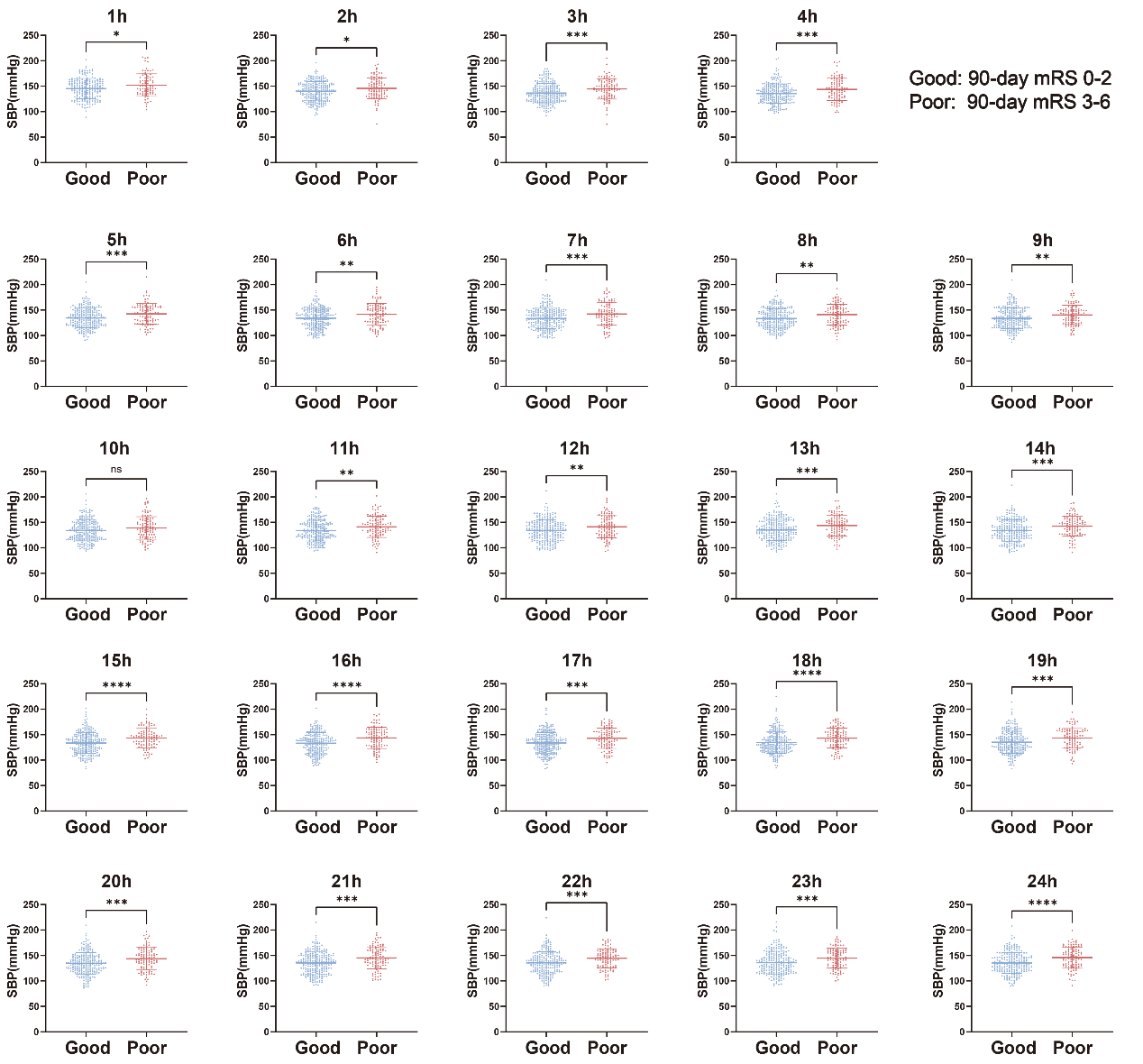


# Figure S2: Distribution of SBP at 24 time points after thrombolysis in the 90-day mRS 0-2 and 3-6 groups.

If the data from both groups follow a normal distribution, a t-test is employed for statistical analysis. However, if any of the groups deviate from a normal distribution, the Mann-Whitney U test is used as an alternative. Statistical significance is denoted as ****p < 0.0001, ***p < 0.001, **p < 0.01, *p < 0.05, and non-significant (not significant) as ns (p ≥ 0.05).


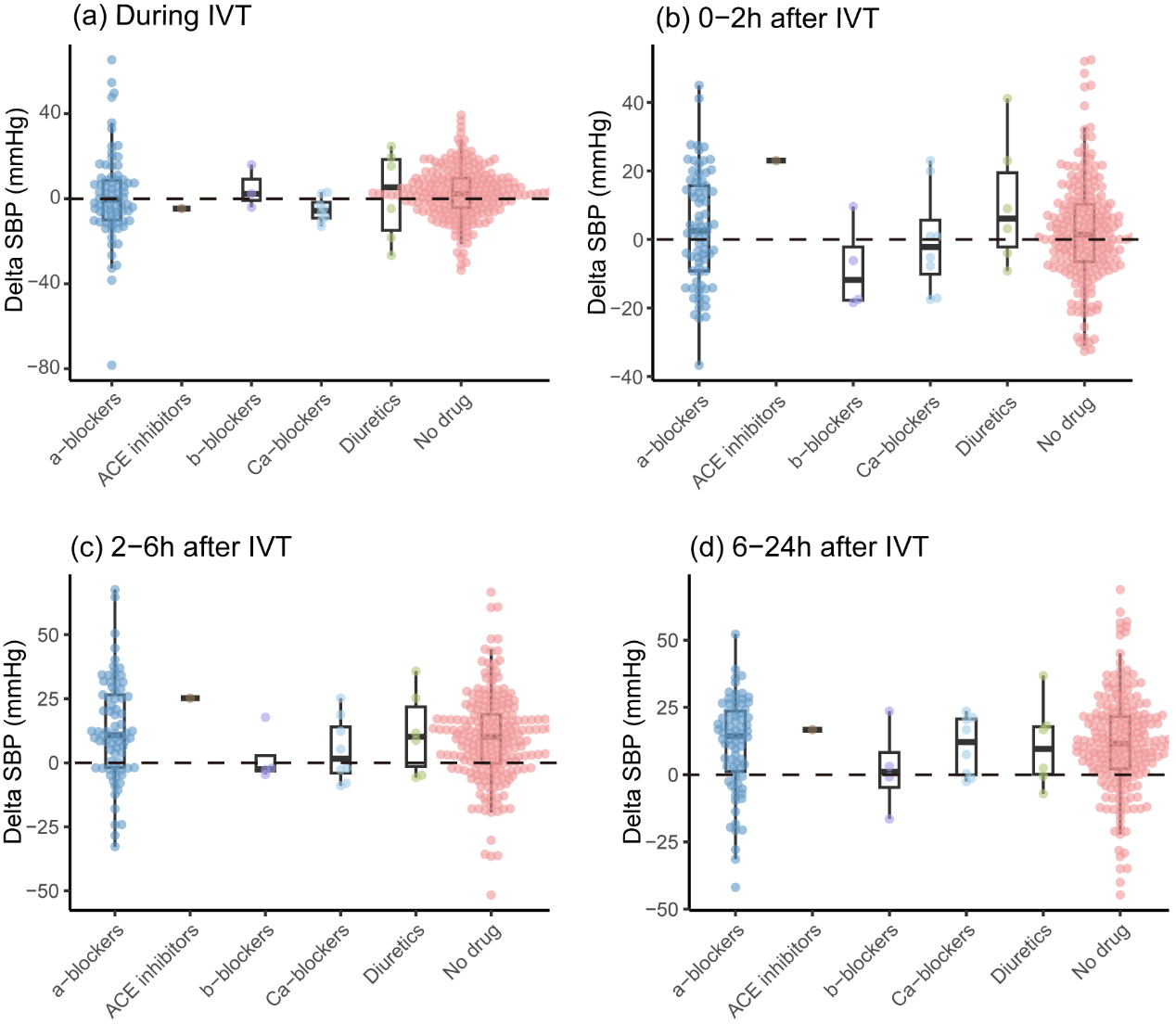


# Figure S3 Box plots of Delta SBP during IVT and different time periods after IVT, stratified by the classes of antihypertensives


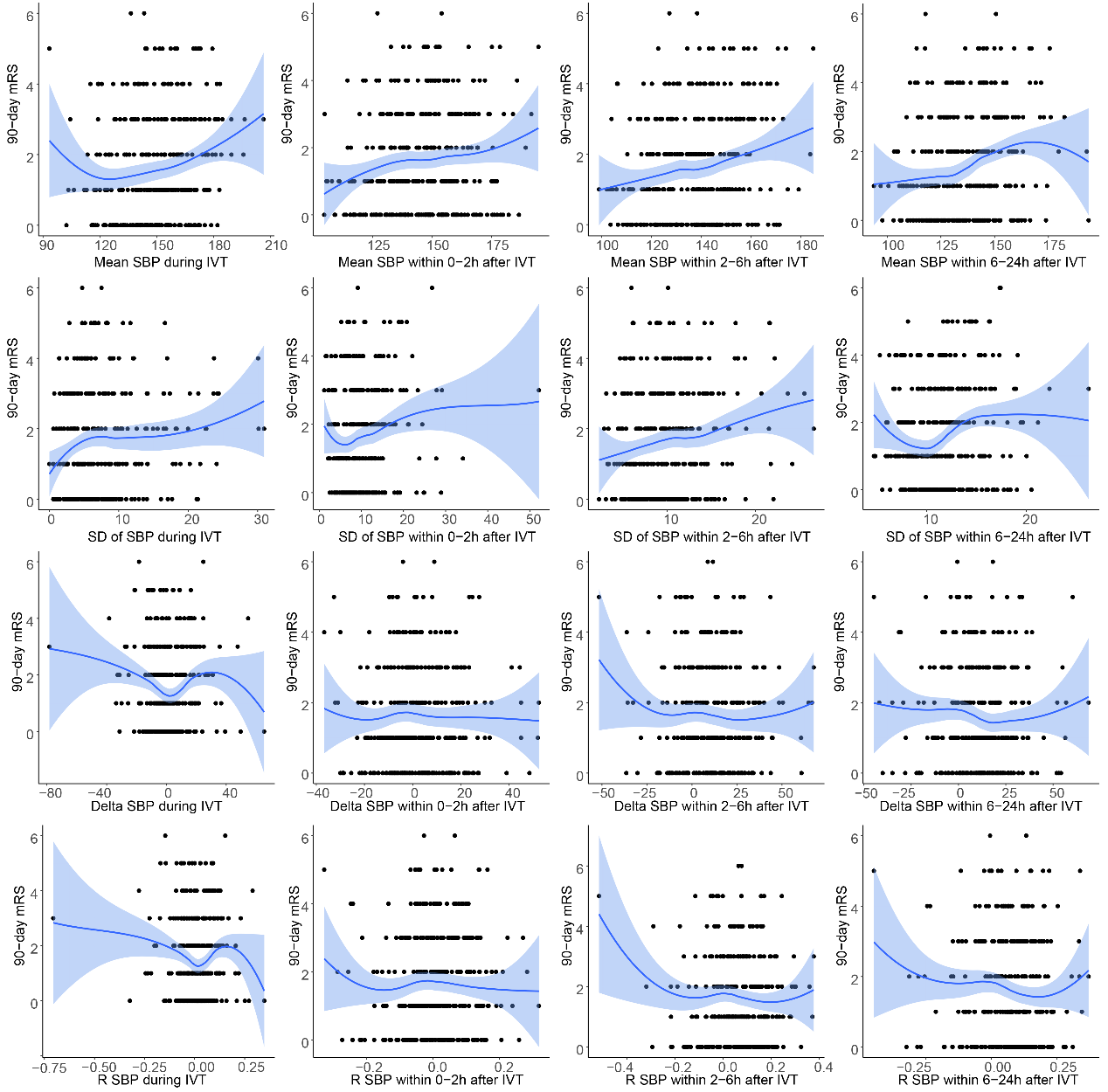


# Figure S4: LOESS plot for association between SBP parameters and ordinal 90-day mRS.

Abbreviations: IVT, intravenous thrombolysis; mRS, modified Rankin Scale; SBP, systolic blood pressure; SD, Standard Deviation; Delta SBP, absolute decrease of systolic blood pressure; R SBP, relative decrease of systolic blood pressure

**
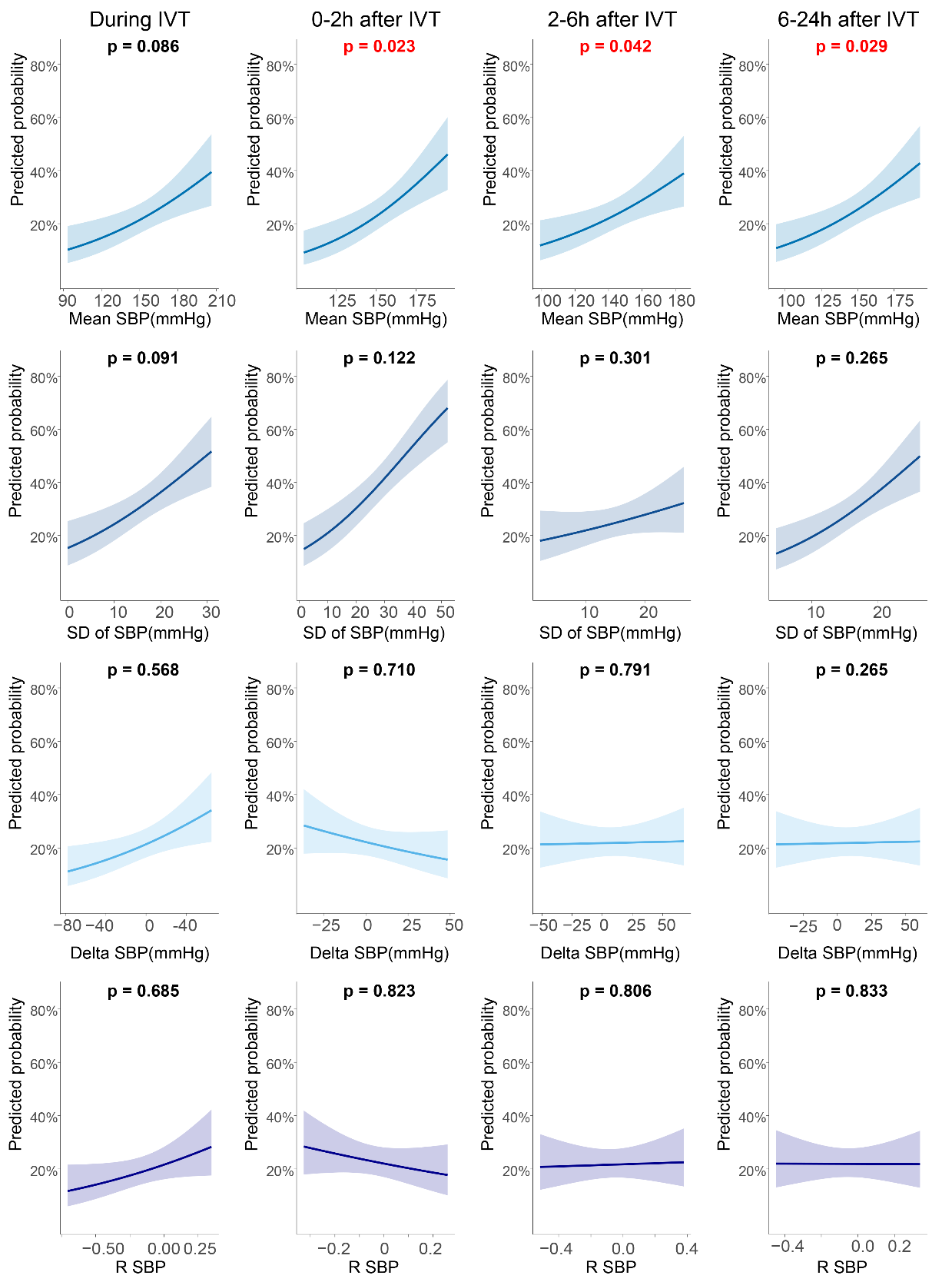
**

# Figure S5: Predicted probability of SBP parameters during IVT and different time periods after IVT on 90-day mRS 3-6.

The analysis was adjusted for age, sex, admission NIHSS, history of atrial fibrillation, history of diabetes mellitus, history of stroke, smoking status. Abbreviations: IVT, intravenous thrombolysis; mRS, modified Rankin Scale


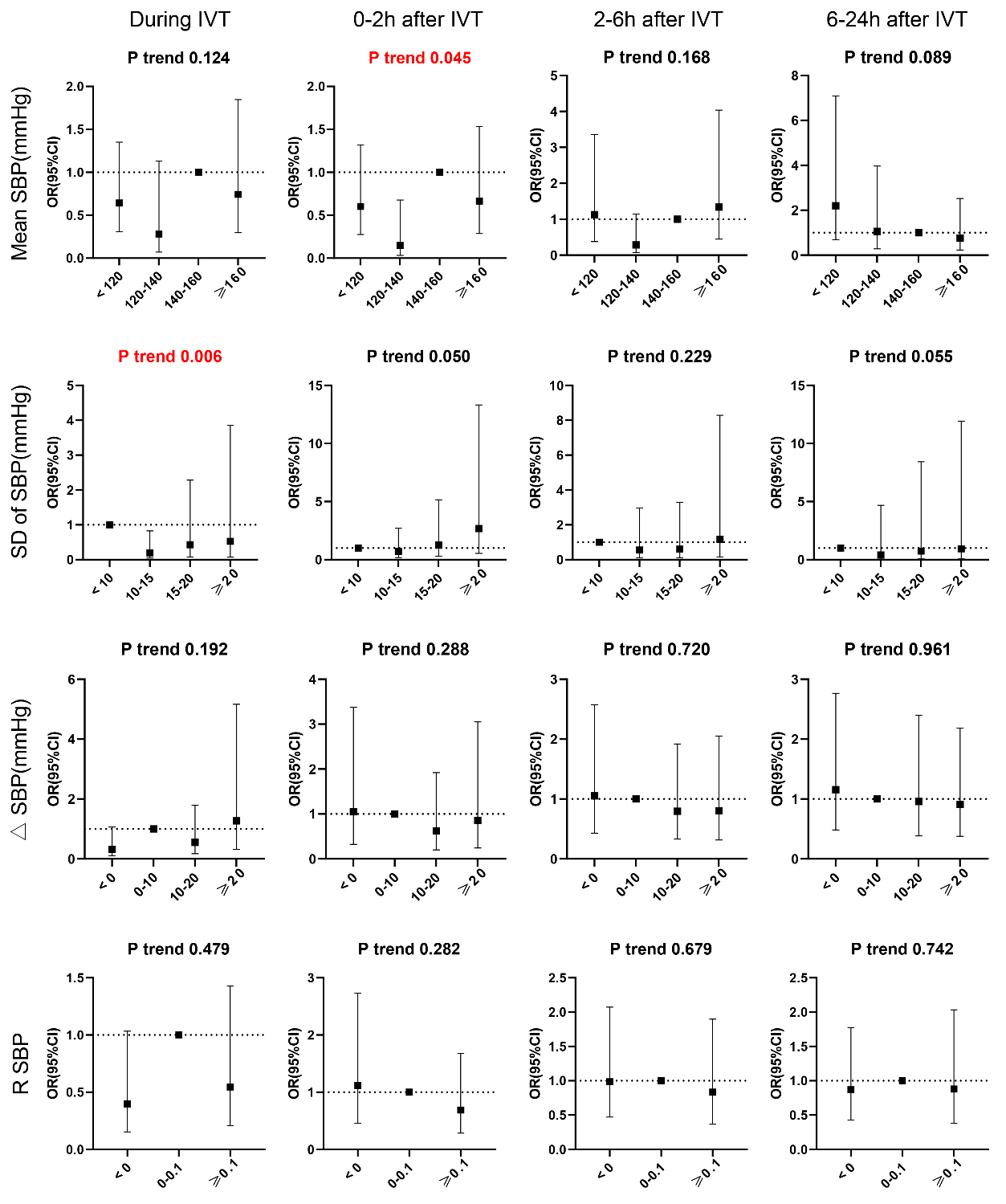


# Figure S6: Association between categorized SBP parameters during IVT and different time periods after IVT and 90-day mRS 2-6.

The odds ratio (OR) and 95% confidence interval (CI) were calculated for each 10mmHg increase in mean SBP and each 10% increase in R SBP. The analysis was adjusted for age, sex, admission NIHSS, history of atrial fibrillation, history of diabetes mellitus, history of stroke, smoking status. Abbreviations: IVT, intravenous thrombolysis; SBP, systolic blood pressure; SD, Standard Deviation; △SBP, absolute decrease of systolic blood pressure; R SBP, relative decrease of systolic blood pressure


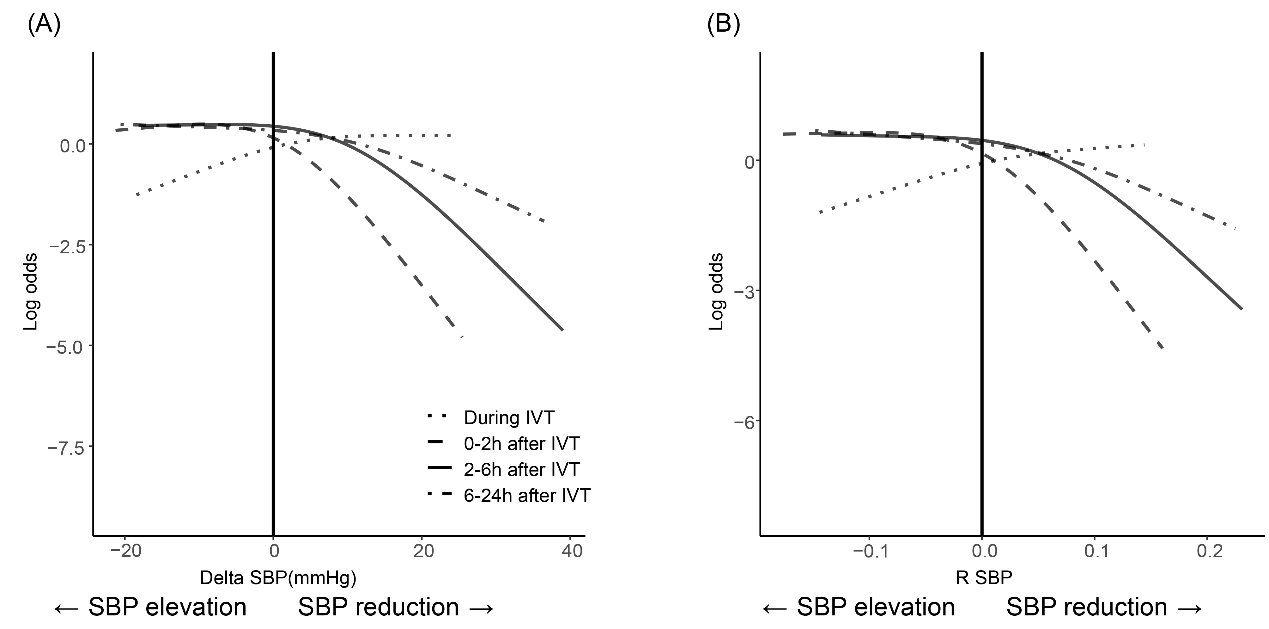


# Figure S7: Restricted cubic spline models of reduction of SBP during IVT and different time periods after IVT and any ICH within 24h after IVT.

The log odds were calculated for each 10% increase in R SBP. The analysis was adjusted for age, sex, admission NIHSS, history of atrial fibrillation, history of diabetes mellitus, history of stroke, smoking status. Abbreviations: IVT, intravenous thrombolysis; SBP, systolic blood pressure; Delta SBP, absolute decrease of systolic blood pressure; R SBP, relative decrease of systolic blood pressure


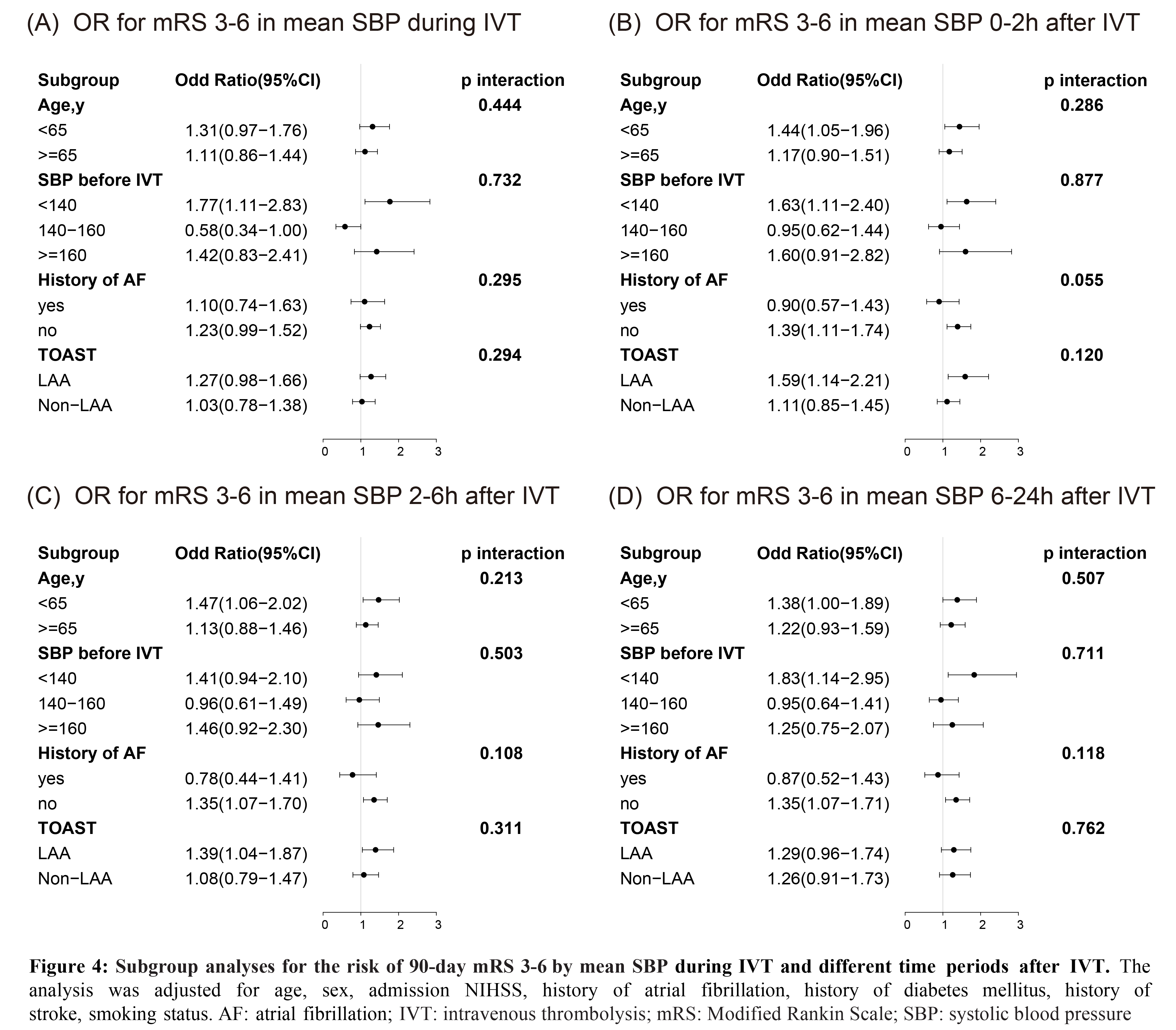


# Figure S8: Subgroup analyses for the risk of 90-day mRS 3-6 by mean SBP during IVT and different time periods after IVT.

The analysis was adjusted for age, sex, admission NIHSS, history of atrial fibrillation, history of diabetes mellitus, history of stroke, smoking status. Abbreviations: AF, atrial fibrillation; IVT, intravenous thrombolysis; mRS, modified Rankin Scale; SBP, systolic blood pressure

# Table S1. Decreases in SBP in IVT-treated patients with and without blood pressure intervention

|  | **Whole (n=340)** | **BP intervention (n=254)** | **No intervention (n=86)** | ***P*** |
| --- | --- | --- | --- | --- |
| **△SBP during IVT** | 2.3(-5.7,9.7) | -0.5(-8.7,8.9) | 2.3(-4.2,9.7) | 0.125 |
| **△SBP t0-2h** | 1.6(-7.0,11.8) | 2.4(-8.8,14.9) | 1.4(-6.5,10.2) | 0.706 |
| **△SBP t2-6h** | 10.1(-1.1,20.9) | 10.5(-2.1,25.7) | 10.1(-0.2,18.8) | 0.433 |
| **△SBP t6-24h** | 11.9(1.9,21.7) | 13.2(-0.7,22.3) | 11.5(2.2,21.6) | 0.899 |

The data are presented as median and interquartile range (IQR), and the differences between the two groups are compared using Mann-Whitney U tests.

# Table S2. Association between 90-day mRS 2-6 and SBP parameters during IVT and different time periods after IVT

| **parameters** | **90-day mRS 2-6** | | | |
| --- | --- | --- | --- | --- |
|  | **unadjusted** | | **adjusted** | |
|  | **OR** | **P** | **OR** | **P** |
| **SBP before IVT**† | 1.21(1.07-1.37) | 0.002 | 1.22(1.03-1.45) | 0.023 |
| **SBP during IVT** |  |  |  |  |
| mean † | 1.24(1.09-1.40) | 0.001 | 1.23(1.03-1.46) | 0.021 |
| SD | 1.07(1.03-1.12) | 0.002 | 1.08(1.02-1.15) | 0.005 |
| △SBP | 1.00(0.98-1.02) | 0.904 | 1.00(0.98-1.03) | 0.893 |
| R SBP‡ | 0.98(0.78-1.23) | 0.839 | 1.01(0.70-1.46) | 0.944 |
| **SBP after IVT**† | 1.22(1.08-1.37) | 0.002 | 1.21(1.02-1.45) | 0.030 |
| **SBP t0-2h** |  |  |  |  |
| mean † | 1.22(1.07-1.39) | 0.003 | 1.21(1.01-1.44) | 0.035 |
| SD | 1.06(1.02-1.11) | 0.005 | 1.04(0.99-1.09) | 0.152 |
| △SBP | 1.01(0.99-1.02) | 0.546 | 1.00(0.98-1.03) | 0.811 |
| R SBP‡ | 1.05(0.84-1.31) | 0.680 | 1.06(0.76-1.47) | 0.741 |
| **SBP t2-6h** |  |  |  |  |
| mean† | 1.26(1.10-1.44) | 0.001 | 1.15(0.96-1.37) | 0.141 |
| SD | 1.09(1.03-1.15) | 0.002 | 1.04(0.97-1.12) | 0.237 |
| △SBP | 1.00(0.99-1.01) | 0.834 | 1.01(0.99-1.03) | 0.321 |
| R SBP‡ | 0.97(0.80-)1.18 | 0.770 | 1.12(0.84-1.50) | 0.436 |
| **SBP t6-24h** |  |  |  |  |
| mean† | 1.36(1.19-1.55) | <0.001 | 1.27(1.06-1.53) | 0.012 |
| SD | 1.12(1.05-1.20) | 0.001 | 1.08(0.99-1.19) | 0.077 |
| △SBP | 1.00(0.98-1.01) | 0.493 | 1.00(0.98-1.02) | 0.899 |
| R SBP‡ | 0.89(0.74-1.08) | 0.249 | 1.00(0.75-1.32) | 0.982 |

† Odds ratio (OR) and 95%CI per 10mmHg increase

‡ Odds ratio (OR) and 95%CI per 10% increase

Adjusted for age, sex, admission NIHSS, history of atrial fibrillation, history of diabetes mellitus, history of stroke, smoking status. Abbreviations: ICH: Intracerebral Hemorrhage; IVT: intravenous thrombolysis; mRS: Modified Rankin Scale; OR: odds ratio; SBP: systolic blood pressure; SD: Standard Deviation.

# Table S3. Association between outcomes and DBP parameters during IVT and different time periods after IVT

| **parameters** | **90-day mRS 3-6** | | **90-day mRS 2-6** | | **Any ICH within 24h** | |
| --- | --- | --- | --- | --- | --- | --- |
|  | **OR** | **P** | **OR** | **P** | **OR** | **P** |
| **DBP before IVT**† | 1.15(0.89-1.49) | 0.298 | 1.31(1.02-1.68) | 0.032 | 0.74(0.52-1.06) | 0.101 |
| **DBP during IVT** |  |  |  |  |  |  |
| mean † | 1.21(0.92-1.59) | 0.165 | 1.41(1.09-1.82) | 0.010 | 0.54(0.36-0.81) | 0.003 |
| SD | 1.00(0.94-1.07) | 0.914 | 1.06(1.00-1.12) | 0.041 | 1.00(0.92-1.09) | 0.962 |
| △DBP | 1.00(0.96-1.04) | 0.976 | 1.00(0.96-1.03) | 0.772 | 1.05(1.00-1.11) | 0.045 |
| R DBP‡ | 0.99(0.72-1.38) | 0.959 | 0.96(0.70-1.32) | 0.803 | 1.69(1.04-2.76) | 0.034 |
| **DBP after IVT**† | 1.35(1.03-1.78) | 0.031 | 1.42(1.10-1.84) | 0.007 | 0.74(0.51-1.07) | 0.108 |
| **DBP t0-2h** |  |  |  |  |  |  |
| mean † | 1.37(1.03-1.83) | 0.033 | 1.35(1.05-1.74) | 0.019 | 0.72(0.49-1.07) | 0.103 |
| SD | 1.02(0.94-1.10) | 0.696 | 1.02(0.95-1.10) | 0.592 | 0.97(0.87-1.07) | 0.500 |
| △DBP | 1.00(0.97-1.04) | 0.938 | 1.01(0.98-1.05) | 0.466 | 0.99(0.95-1.04) | 0.764 |
| R DBP‡ | 1.00(0.74-1.34) | 0.978 | 1.08(0.82-1.43) | 0.589 | 0.91(0.62-1.35) | 0.651 |
| **DBP t2-6h** |  |  |  |  |  |  |
| mean† | 1.45(1.07-1.96) | 0.016 | 1.48(1.13-1.95) | 0.005 | 0.95(0.63-1.44) | 0.820 |
| SD | 0.99(0.90-1.08) | 0.782 | 1.01(0.93-1.10) | 0.818 | 1.01(0.88-1.15) | 0.926 |
| △DBP | 1.01(0.97-1.04) | 0.734 | 1.01(0.98-1.04) | 0.495 | 0.96(0.92-1.01) | 0.097 |
| R DBP‡ | 1.03(0.78-1.36) | 0.843 | 1.03(0.80-1.33) | 0.793 | 0.72(0.50-1.05) | 0.088 |
| **DBP t6-24h** |  |  |  |  |  |  |
| mean† | 1.33(0.98-1.79) | 0.064 | 1.50(1.14-1.98) | 0.004 | 0.95(0.63-1.42) | 0.797 |
| SD | 1.04(0.92-1.18) | 0.515 | 1.05(0.94-1.18) | 0.397 | 0.94(0.80-1.11) | 0.479 |
| △DBP | 1.02(0.99-1.05) | 0.206 | 1.01(0.99-1.04) | 0.349 | 0.98(0.94-1.02) | 0.230 |
| R DBP‡ | 1.16(0.89-1.52) | 0.272 | 1.08(0.85-1.39) | 0.526 | 0.82(0.58-1.15) | 0.243 |

† Odds ratio (OR) and 95%CI per 10mmHg increase

‡ Odds ratio (OR) and 95%CI per 10% increase

The above model is adjusted for age, sex, admission NIHSS, history of atrial fibrillation, history of diabetes mellitus, history of stroke, smoking status. Abbreviations: DBP: diastolic blood pressure; ICH: Intracerebral Hemorrhage; IVT: intravenous thrombolysis; mRS: Modified Rankin Scale; OR: odds ratio; SD: Standard Deviation

# Table S4. Association between outcomes and MAP parameters during IVT and different time periods after IVT.

| **parameters** | **90-day mRS 3-6** | | **90-day mRS 2-6** | | **Any ICH within 24h** | |
| --- | --- | --- | --- | --- | --- | --- |
|  | **OR** | **P** | **OR** | **P** | **OR** | **P** |
| **MAP before IVT**† | 1.23(0.95-1.58) | 0.115 | 1.35(1.06-1.72) | 0.016 | 0.72(0.51-1.02) | 0.064 |
| **MAP during IVT** |  |  |  |  |  |  |
| mean † | 1.25(0.96-1.62) | 0.092 | 1.39(1.09-1.77) | 0.008 | 0.52(0.35-0.78) | 0.002 |
| SD | 1.04(0.97-1.12) | 0.299 | 1.09(1.02-1.16) | 0.014 | 1.00(0.91-1.11) | 0.935 |
| △MAP | 1.01(0.97-1.05) | 0.747 | 1.00(0.96-1.03) | 0.900 | 1.06(1.01-1.11) | 0.024 |
| R MAP‡ | 1.07(0.69-1.65) | 0.774 | 0.99(0.66-1.49) | 0.953 | 2.08(1.14-3.79) | 0.017 |
| **MAP after IVT**† | 1.37(1.05-1.78) | 0.021 | 1.41(1.10-1.80) | 0.007 | 0.66(0.46-0.95) | 0.027 |
| **MAP t0-2h** |  |  |  |  |  |  |
| mean † | 1.41(1.07-1.87) | 0.016 | 1.36(1.06-1.73) | 0.014 | 0.75(0.51-1.10) | 0.140 |
| SD | 1.03(0.96-1.10) | 0.474 | 1.04(0.97-1.12) | 0.275 | 0.93(0.83-1.04) | 0.194 |
| △MAP | 1.00(0.96-1.04) | 0.930 | 1.01(0.98-1.04) | 0.540 | 0.96(0.92-1.01) | 0.154 |
| R MAP‡ | 0.99(0.67-1.46) | 0.968 | 1.10(0.78-1.57) | 0.579 | 0.66(0.39-1.11) | 0.115 |
| **MAP t2-6h** |  |  |  |  |  |  |
| mean† | 1.43(1.08-1.91) | 0.013 | 1.37(1.06-1.77) | 0.015 | 0.95(0.64-1.40) | 0.790 |
| SD | 1.02(0.92-1.12) | 0.762 | 1.03(0.94-1.13) | 0.561 | 0.97(0.84-1.11) | 0.628 |
| △MAP | 1.01(0.98-1.04) | 0.736 | 1.01(0.99-1.04) | 0.372 | 0.95(0.91-0.99) | 0.025 |
| R MAP‡ | 1.04(0.75-1.45) | 0.803 | 1.09(0.81-1.47) | 0.584 | 0.60(0.38-0.94) | 0.026 |
| **MAP t6-24h** |  |  |  |  |  |  |
| mean† | 1.36(1.03-1.81) | 0.030 | 1.46(1.13-1.89) | 0.004 | 0.89(0.61-1.30) | 0.539 |
| SD | 1.08(0.95-1.22) | 0.245 | 1.12(0.99-1.26) | 0.062 | 0.88(0.73-1.06) | 0.170 |
| △MAP | 1.01(0.99-1.04) | 0.350 | 1.01(0.98-1.04) | 0.520 | 0.97(0.93-1.01) | 0.125 |
| R MAP‡ | 1.14(0.83-1.56) | 0.432 | 1.06(0.79-1.41) | 0.711 | 0.73(0.49-1.09) | 0.120 |

† Odds ratio (OR) and 95%CI per 10mmHg increase

‡ Odds ratio (OR) and 95%CI per 10% increase

The above model is adjusted for age, sex, admission NIHSS, history of atrial fibrillation, history of diabetes mellitus, history of stroke, smoking status. Abbreviations: ICH: Intracerebral Hemorrhage; IVT: intravenous thrombolysis; MAP: mean arterial pressure; mRS: Modified Rankin Scale; OR: odds ratio; SD: Standard Deviation

# Table S5. Association between categorical mean systolic blood pressure with 90-day mRS 3-6

| 90-day mRS 3-6 | | | |
| --- | --- | --- | --- |
|  |  | OR(95%CI) | P trend |
| Mean SBP during IVT | <120mmHg | 0.76(0.35-1.67) | 0.141 |
|  | 120-140mmHg | 0.49(0.11-2.16) |  |
|  | 140-160mmHg | 1.0 |  |
|  | ≥160mmHg | 0.50(0.18-1.40) |  |
| Mean SBP within 0-2h after IVT | <120mmHg | 0.51(0.22-1.17) | 0.012 |
|  | 120-140mmHg | 0.12(0.02-0.75) |  |
|  | 140-160mmHg | 1.0 |  |
|  | ≥160mmHg | 0.44(0.18-1.09) |  |
| Mean SBP within 2-6h after IVT | <120mmHg | 0.57(0.19-1.71) | 0.022 |
|  | 120-140mmHg | 0.19(0.04-0.82) |  |
|  | 140-160mmHg | 1.0 |  |
|  | ≥160mmHg | 0.44(0.15-1.35) |  |
| Mean SBP within 6-24h after IVT | <120mmHg | 2.42(0.74-7.95) | 0.110 |
|  | 120-140mmHg | 1.14(0.28-4.71) |  |
|  | 140-160mmHg | 1.0 |  |
|  | ≥160mmHg | 0.71(0.20-2.48) |  |

Odds ratio (OR) and 95%CI per 10mmHg increase

The above model is adjusted for age, sex, admission NIHSS, history of atrial fibrillation, history of diabetes mellitus, history of stroke, smoking status. Abbreviations: IVT: intravenous thrombolysis; mRS: Modified Rankin Scale; SBP: systolic blood pressure.

# Table S6. Subgroup analyses for the risk of mRS 3-6 at 90 days by mean SBP during IVT and different time periods after IVT

| **Subgroup** | **Odds Ratio(95%CI)** | **p value** | **p interaction** |
| --- | --- | --- | --- |
| **Mean SBP during IVT** |  |  |  |
| **Age,y** |  |  | 0.444 |
| ＜65 | 1.31(0.97-1.76) | 0.076 |  |
| ≥65 | 1.11(0.86-1.44) | 0.428 |  |
| **SBP before IVT** |  |  | 0.732 |
| ＜140 | 1.77(1.11-2.83) | 0.018 |  |
| 140-160 | 0.58(0.34-1.00) | 0.049 |  |
| ≥160 | 1.42(0.83-2.41) | 0.201 |  |
| **History of AF** |  |  | 0.295 |
| yes | 1.10(0.74-1.63) | 0.629 |  |
| no | 1.23(0.99-1.52) | 0.057 |  |
| **TOAST** |  |  | 0.294 |
| LAA | 1.27(0.98-1.66) | 0.074 |  |
| Non-LAA | 1.03(0.78-1.38) | 0.826 |  |
| **Mean SBP within 0-2h after IVT** |  |  |  |
| **Age,y** |  |  | 0.286 |
| ＜65 | 1.44(1.05-1.96) | 0.024 |  |
| ≥65 | 1.17(0.90-1.51) | 0.241 |  |
| **SBP before IVT** |  |  | 0.877 |
| ＜140 | 1.63(1.11-2.40) | 0.014 |  |
| 140-160 | 0.95(0.62-1.44) | 0.797 |  |
| ≥160 | 1.60(0.91-2.82) | 0.104 |  |
| **History of AF** |  |  | 0.055 |
| yes | 0.90(0.57-1.43) | 0.659 |  |
| no | 1.39(1.11-1.74) | 0.004 |  |
| **TOAST** |  |  | 0.120 |
| LAA | 1.59(1.14-2.21) | 0.006 |  |
| Non-LAA | 1.11(0.85-1.45) | 0.434 |  |
| **Mean SBP within 2-6h after IVT** |  |  |  |
| **Age,y** |  |  | 0.213 |
| ＜65 | 1.47(1.06-2.02) | 0.020 |  |
| ≥65 | 1.13(0.88-1.46) | 0.337 |  |
| **SBP before IVT** |  |  | 0.503 |
| ＜140 | 1.41(0.94-2.10) | 0.097 |  |
| 140-160 | 0.96(0.61-1.49) | 0.844 |  |
| ≥160 | 1.46(0.92-2.30) | 0.110 |  |
| **History of AF** |  |  | 0.108 |
| yes | 0.78(0.44-1.41) | 0.413 |  |
| no | 1.35(1.07-1.70) | 0.010 |  |
| **TOAST** |  |  | 0.311 |
| LAA | 1.39(1.04-1.87) | 0.028 |  |
| **Subgroup** | **Odds Ratio(95%CI)** | **p value** | **p interaction** |
| Non-LAA | 1.08(0.79-1.47) | 0.628 |  |
| **Mean SBP within 2-6h after IVT** |  |  |  |
| **Age,y** |  |  | 0.507 |
| ＜65 | 1.38(1.00-1.89) | 0.048 |  |
| ≥65 | 1.22(0.93-1.59) | 0.152 |  |
| **SBP before IVT** |  |  | 0.711 |
| ＜140 | 1.83(1.14-2.95) | 0.013 |  |
| 140-160 | 0.95(0.64-1.41) | 0.791 |  |
| ≥160 | 1.25(0.75-2.07) | 0.386 |  |
| **History of AF** |  |  | 0.118 |
| yes | 0.87(0.52-1.43) | 0.571 |  |
| no | 1.35(1.07-1.71) | 0.011 |  |
| **TOAST** |  |  | 0.762 |
| LAA | 1.29(0.96-1.74) | 0.089 |  |
| Non-LAA | 1.26(0.91-1.73) | 0.160 |  |

Odds ratio (OR) and 95%CI per 10mmHg increase

The above model is adjusted for age, sex, admission NIHSS, history of atrial fibrillation, history of diabetes mellitus, history of stroke, smoking status. Abbreviations: AF: atrial fibrillation; IVT: intravenous thrombolysis; LAA: large artery atherosclerosis; SBP: systolic blood pressure.

# Table S7. Subgroup analyses for the risk of mRS 2-6 at 90 days by mean SBP during IVT and different time periods after IVT

| **Subgroup** | **Odds Ratio(95%CI)** | **p value** | **p interaction** |
| --- | --- | --- | --- |
| **Mean SBP during IVT** |  |  |  |
| **Age,y** |  |  | 0.150 |
| <65 | 1.48(1.11-1.97) | 0.008 |  |
| ≥65 | 1.09(0.85-1.40) | 0.483 |  |
| **SBP before IVT** |  |  | 0.612 |
| <140 | 1.77(1.06-2.96) | 0.029 |  |
| 140-160 | 0.63(0.42-0.95) | 0.027 |  |
| ≥160 | 1.76(0.98-3.17) | 0.060 |  |
| **History of AF** |  |  | 0.574 |
| yes | 1.11(0.74-1.19) | 0.654 |  |
| no | 1.25(1.03-1.52) | 0.023 |  |
| **TOAST** |  |  | 0.649 |
| LAA | 1.28(1.01-1.62) | 0.038 |  |
| Non-LAA | 1.24(0.94-1.65) | 0.130 |  |
| **Mean SBP within 0-2h after IVT** |  |  |  |
| **Age,y** |  |  | 0.759 |
| <65 | 1.26(0.98-1.62) | 0.073 |  |
| ≥65 | 1.22(0.95-1.56) | 0.125 |  |
| **SBP before IVT** |  |  | 0.133 |
| <140 | 1.49(1.03-2.14) | 0.032 |  |
| 140-160 | 1.06(0.75-1.50) | 0.740 |  |
| ≥160 | 0.90(0.59-1.36) | 0.608 |  |
| **History of AF** |  |  | 0.466 |
| yes | 0.98(0.62-1.56) | 0.945 |  |
| no | 1.25(1.03-1.52) | 0.028 |  |
| **TOAST** |  |  | 0.665 |
| LAA | 1.26(0.96-1.66) | 0.097 |  |
| Non-LAA | 1.21(0.95-1.54) | 0.131 |  |
| **Mean SBP within 2-6h after IVT** |  |  |  |
| **Age,y** |  |  | 0.385 |
| <65 | 1.28(0.99-1.66) | 0.061 |  |
| ≥65 | 1.11(0.86-1.42) | 0.423 |  |
| **SBP before IVT** |  |  | 0.403 |
| <140 | 1.28(0.87-1.87) | 0.209 |  |
| 140-160 | 1.07(0.76-1.50) | 0.692 |  |
| ≥160 | 0.85(0.58-1.25) | 0.406 |  |
| **History of AF** |  |  | 0.257 |
| yes | 0.71(0.39-1.26) | 0.240 |  |
| no | 1.21(0.98-1.48) | 0.072 |  |
| **TOAST** |  |  | 0.480 |
| LAA | 1.24(0.95-1.63) | 0.114 |  |
| **Subgroup** | **Odds Ratio(95%CI)** | **p value** | **p interaction** |
| Non-LAA | 1.09(0.84-1.43) | 0.509 |  |
| **Mean SBP within 2-6h after IVT** |  |  |  |
| **Age,y** |  |  | 0.480 |
| <65 | 1.41(1.08-1.85) | 0.012 |  |
| ≥65 | 1.26(0.96-1.65) | 0.090 |  |
| **SBP before IVT** |  |  | 0.323 |
| <140 | 1.58(1.02-2.45) | 0.041 |  |
| 140-160 | 1.14(0.85-1.54) | 0.383 |  |
| ≥160 | 0.94(0.58-1.51) | 0.789 |  |
| **History of AF** |  |  | 0.644 |
| yes | 1.07(0.66-1.71) | 0.794 |  |
| no | 1.30(1.05-1.60) | 0.014 |  |
| **TOAST** |  |  | 0.530 |
| LAA | 1.18(0.91-1.53) | 0.218 |  |
| Non-LAA | 1.38(1.04-1.83) | 0.024 |  |

Odds ratio (OR) and 95%CI per 10mmHg increase

The above model is adjusted for age, sex, admission NIHSS, history of atrial fibrillation, history of diabetes mellitus, history of stroke, smoking status. Abbreviations: AF: atrial fibrillation; IVT: intravenous thrombolysis; LAA: large artery atherosclerosis; SBP: systolic blood pressure.

# Table S8. Subgroup analyses for the risk of any ICH within 24h by △SBP and R SBP during different time periods after IVT

| **Subgroup** | **Odds Ratio(95%CI)** | **p value** | **p interaction** |
| --- | --- | --- | --- |
| **△SBP within 0-2h after IVT** |  |  |  |
| **Age,y** |  |  | 0.465 |
| <65 | 0.91(0.82-1.01) | 0.066 |  |
| ≥65 | 0.96(0.91-1.00) | 0.065 |  |
| **SBP before IVT** |  |  | 0.664 |
| <140 | 0.95(0.87-1.04) | 0.287 |  |
| 140-160 | 0.94(0.88-1.00) | 0.057 |  |
| ≥160 | 0.97(0.88-1.06) | 0.486 |  |
| **History of AF** |  |  | 0.976 |
| yes | 0.94(0.88-1.01) | 0.082 |  |
| no | 0.94(0.89-0.99) | 0.028 |  |
| **TOAST** |  |  | 0.329 |
| LAA | 0.93(0.87-0.99) | 0.034 |  |
| Non-LAA | 0.96(0.91-1.01) | 0.152 |  |
| **△SBP within 2-6h after IVT** |  |  |  |
| **Age,y** |  |  | 0.885 |
| <65 | 0.96(0.89-1.03) | 0.261 |  |
| ≥65 | 0.97(0.93-1.00) | 0.048 |  |
| **SBP before IVT** |  |  | 0.225 |
| <140 | 0.98(0.91-1.05) | 0.522 |  |
| 140-160 | 0.96(0.92-1.01) | 0.110 |  |
| ≥160 | 0.93(0.86-1.02) | 0.108 |  |
| **History of AF** |  |  | 0.630 |
| yes | 0.94(0.89-1.00) | 0.049 |  |
| no | 0.97(0.93-1.01) | 0.120 |  |
| **TOAST** |  |  | 0.792 |
| LAA | 0.96(0.91-1.02) | 0.190 |  |
| Non-LAA | 0.96(0.92-1.00) | 0.051 |  |
| **R SBP within 0-2h after IVT**‡ |  |  |  |
| **Age,y** |  |  | 0.326 |
| <65 | 0.25(0.06-0.95) | 0.042 |  |
| ≥65 | 0.55(0.28-1.09) | 0.085 |  |
| **SBP before IVT** |  |  | 0.689 |
| <140 | 0.46(0.14-1.43) | 0.178 |  |
| 140-160 | 0.42(0.16-1.06) | 0.066 |  |
| ≥160 | 0.60(0.14-2.57) | 0.491 |  |
| **History of AF** |  |  | 0.874 |
| yes | 0.43(0.16-1.14) | 0.088 |  |
| no | 0.39(0.18-0.83) | 0.015 |  |
| **TOAST** |  |  | 0.207 |
| LAA | 0.32(0.13-0.81) | 0.017 |  |
| Non-LAA | 0.62(0.31-1.23) | 0.170 |  |
| **R SBP within 2-6h after IVT**‡ |  |  |  |
| **Age,y** |  |  | 0.876 |
| <65 | 0.53(0.19-1.51) | 0.236 |  |
| ≥65 | 0.62(0.38-1.03) | 0.066 |  |
| **SBP before IVT** |  |  | 0.209 |
| <140 | 0.70(0.30-1.63) | 0.409 |  |
| 140-160 | 0.62(0.32-1.20) | 0.155 |  |
| ≥160 | 0.35(0.10-1.22) | 0.099 |  |
| **History of AF** |  |  | 0.605 |
| yes | 0.45(0.19-1.04) | 0.060 |  |
| no | 0.65(0.36-1.17) | 0.148 |  |
| **TOAST** |  |  | 0.733 |
| LAA | 0.59(0.26-1.33) | 0.201 |  |
| Non-LAA | 0.60(0.35-1.03) | 0.063 |  |

‡Odds ratio (OR) and 95%CI per 10% increase

The above model is adjusted for age, sex, admission NIHSS, history of atrial fibrillation, history of diabetes mellitus, history of stroke, smoking status. Abbreviations: AF: atrial fibrillation; IVT: intravenous thrombolysis; LAA: large artery atherosclerosis; SBP: systolic blood pressure.

# Table S9. Association between outcomes and SBP parameters during IVT and different time periods after IVT (timepoints of blood pressure measurements >= 35)

| **parameters** | **90-day mRS 3-6** | | **90-day mRS 2-6** | | **Any ICH within 24h** | |
| --- | --- | --- | --- | --- | --- | --- |
|  | **OR** | **P** | **OR** | **P** | **OR** | **P** |
| **SBP before IVT†** | 1.22(0.99-1.51) | 0.060 | 1.20(0.99-1.45) | 0.060 | 0.81(0.61-1.08) | 0.154 |
| **SBP during IVT** |  |  |  |  |  |  |
| mean † | 1.25(1.00-1.55) | 0.050 | 1.21(1.00-1.47) | 0.057 | 0.63(0.45-0.88) | 0.006 |
| SD | 1.08(1.01-1.15) | 0.025 | 1.08(1.02-1.15) | 0.014 | 1.01(0.91-1.12) | 0.849 |
| △SBP | 1.00(0.97-1.03) | 0.852 | 1.01(0.98-1.04) | 0.702 | 1.05(1.00-1.10) | 0.038 |
| R SBP‡ | 0.97(0.60-1.57) | 0.909 | 1.04(0.68-1.60) | 0.857 | 2.24(1.01-4.94) | 0.046 |
| **SBP after IVT†** | 1.18(0.95-1.45) | 0.132 | 1.18(0.97-1.43) | 0.097 | 0.67(0.49-0.91) | 0.011 |
| **SBP t0-2h** |  |  |  |  |  |  |
| mean † | 1.29(1.01-1.63) | 0.038 | 1.20(0.98-1.48) | 0.075 | 0.88(0.64-1.23) | 0.461 |
| SD | 1.11(1.03-1.20) | 0.005 | 1.11(1.03-1.20) | 0.007 | 0.92(0.83-1.02) | 0.118 |
| △SBP | 0.99(0.96-1.02) | 0.420 | 1.00(0.97-1.02) | 0.832 | 0.94(0.89-0.98) | 0.007 |
| R SBP‡ | 0.87(0.56-1.33) | 0.509 | 0.98(0.68-1.41) | 0.899 | 0.39(0.21-0.75) | 0.005 |
| **SBP t2-6h** |  |  |  |  |  |  |
| mean† | 1.17(0.93-1.46) | 0.184 | 1.11(0.90-1.36) | 0.330 | 0.97(0.69-1.34) | 0.831 |
| SD | 1.03(0.93-1.13) | 0.583 | 1.04(0.96-1.13) | 0.337 | 0.94(0.81-1.10) | 0.438 |
| △SBP | 1.00(0.98-1.03) | 0.756 | 1.01(0.99-1.03) | 0.422 | 0.94(0.91-0.98) | 0.006 |
| R SBP‡ | 1.04(0.72-1.50) | 0.845 | 1.11(0.80-1.53) | 0.541 | 0.46(0.26-0.81) | 0.007 |
| **SBP t6-24h** |  |  |  |  |  |  |
| mean† | 1.19(0.94-1.50) | 0.145 | 1.23(1.00-1.51) | 0.054 | 0.83(0.59-1.17) | 0.285 |
| SD | 1.17(1.04-1.32) | 0.012 | 1.16(1.04-1.29) | 0.010 | 0.98(0.81-1.18) | 0.808 |
| △SBP | 1.00(0.98-1.03) | 0.843 | 1.00(0.98-1.02) | 0.895 | 0.97(0.93-1.00) | 0.041 |
| R SBP‡ | 1.01(0.70-1.47) | 0.947 | 0.96(0.69-1.34) | 0.828 | 0.58(0.36-0.95) | 0.029 |

† Odds ratio (OR) and 95%CI per 10mmHg increase

‡ Odds ratio (OR) and 95%CI per 10% increase

The above model is adjusted for age, sex, admission NIHSS, history of atrial fibrillation, history of diabetes mellitus, history of stroke, smoking status. Abbreviations: SBP: systolic blood pressure; ICH: Intracerebral Hemorrhage; IVT: intravenous thrombolysis; mRS: Modified Rankin Scale; OR: odds ratio; SD: Standard Deviation

# Table S10. Association between outcomes and SBP parameters during IVT and different time periods after IVT (additionally adjusted for comorbidity)

| **parameters** | **90-day mRS 3-6** | | **90-day mRS 2-6** | | **Any ICH within 24h** | |
| --- | --- | --- | --- | --- | --- | --- |
|  | **OR** | **P** | **OR** | **P** | **OR** | **P** |
| **SBP before IVT†** | 1.23(1.02-1.50) | 0.033 | 1.24(1.03-1.49) | 0.022 | 0.81(0.61-1.06) | 0.127 |
| **SBP during IVT** |  |  |  |  |  |  |
| mean † | 1.20(0.99-1.47) | 0.066 | 1.23(1.02-1.48) | 0.032 | 0.72(0.54-0.94) | 0.020 |
| SD | 1.05(0.99-1.12) | 0.079 | 1.08(1.02-1.14) | 0.011 | 0.96(0.86-1.06) | 0.472 |
| **△**SBP | 1.01(0.98-1.03) | 0.519 | 1.00(0.98-1.03) | 0.713 | 1.02(0.99-1.06) | 0.202 |
| R SBP‡ | 1.09(0.74-1.56) | 0.633 | 1.06(0.72-1.53) | 0.759 | 1.52(0.88-2.81) | 0.168 |
| **SBP after IVT†** | 1.22(1.01-1.49) | 0.044 | 1.08(0.92-1.29) | 0.361 | 0.80(0.64-1.00) | 0.037 |
| **SBP t0-2h** |  |  |  |  |  |  |
| mean † | 1.27(1.03-1.57) | 0.025 | 1.23(1.02-1.49) | 0.036 | 0.96(0.74-1.23) | 0.728 |
| SD | 1.04(0.99-1.10) | 0.125 | 1.04(0.99-1.09) | 0.165 | 0.90(0.81-0.98) | 0.033 |
| **△**SBP | 1.00(0.98-1.02) | 0.903 | 0.99(0.97-1.01) | 0.318 | 0.98(0.95-1.00) | 0.036 |
| R SBP‡ | 0.97(0.66-1.45) | 0.891 | 1.03(0.74-1.46) | 0.846 | 0.50(0.28-0.86) | 0.014 |
| **SBP t2-6h** |  |  |  |  |  |  |
| mean† | 1.24(1.00-1.53) | 0.047 | 1.14(0.95-1.39) | 0.165 | 1.04(0.79-1.38) | 0.763 |
| SD | 1.04(0.96-1.12) | 0.336 | 1.04(0.97-1.12) | 0.297 | 0.93(0.82-1.04) | 0.203 |
| **△**SBP | 1.00(0.99-1.03) | 0.655 | 1.00(0.98-1.02) | 0.908 | 0.98(0.96-1.00) | 0.033 |
| R SBP‡ | 1.07(0.77-1.50) | 0.708 | 1.16(0.86-1.58) | 0.337 | 0.62(0.38-0.95) | 0.035 |
| **SBP t6-24h** |  |  |  |  |  |  |
| mean† | 1.26(1.02-1.56) | 0.034 | 1.27(1.05-1.56) | 0.016 | 0.89(0.67-1.17) | 0.404 |
| SD | 1.05(0.95-1.16) | 0.304 | 1.07(0.98-1.18) | 0.117 | 0.86(0.73-1.00) | 0.064 |
| **△**SBP | 1.00(0.99-1.03) | 0.683 | 0.99(0.98-1.01) | 0.395 | 0.99(0.97-1.01) | 0.211 |
| R SBP‡ | 1.05(0.76-1.46) | 0.783 | 1.02(0.76-1.37) | 0.901 | 0.75(0.50-1.12) | 0.159 |

† Odds ratio (OR) and 95%CI per 10mmHg increase

‡ Odds ratio (OR) and 95%CI per 10% increase

The above model is adjusted for age, sex, admission NIHSS, history of atrial fibrillation, history of diabetes mellitus, history of stroke, smoking status, pulmonary infection, urinary tract infection, deep vein thrombosis in the lower extremities, heart failure, liver dysfunction, renal dysfunction, extracranial hemorrhage, myocardial infarction, symptomatic epilepsy, and post-stroke emotional disturbance. Abbreviations: SBP: systolic blood pressure; ICH: Intracerebral Hemorrhage; IVT: intravenous thrombolysis; mRS: Modified Rankin Scale; OR: odds ratio; SD: Standard Deviation
